# Investigating the Role of Amazonian Mesoscale Wind Patterns and Strength on the Spatial Distribution of Martian Bedrock Exposures

**DOI:** 10.1029/2022JE007496

**Published:** 2022-11-18

**Authors:** C. E. Gary‐Bicas, T. I. Michaels, A. D. Rogers, L. K. Fenton, N. H. Warner, A. C. Cowart

**Affiliations:** ^1^ Department of Geosciences Stony Brook University Stony Brook NY USA; ^2^ Carl Sagan Center SETI Institute Mountain View CA USA; ^3^ Department of Geological Sciences State University of New York at Geneseo Geneseo NY USA

**Keywords:** Mars, bedrock exposures, material properties, atmosphere, erosional processes, mesoscale

## Abstract

The Martian highlands contain Noachian‐aged areally‐extensive (>225 km^2^) bedrock exposures that have been mapped using thermal and visible imaging datasets. Given their age, crater density and impact gardening should have led to the formation of decameter scale layers of regolith that would overlie and bury these outcrops if composed of competent materials like basaltic lavas. However, many of these regions lack thick regolith layers and show clear exposures of bedrock materials with elevated thermal inertia values compared to the global average. Hypothesized reasons for the lack of regolith include: (a) relatively weaker material properties than lavas, where friable materials are comminuted and deflated during wind erosion, (b) long‐term protection from regolith development through burial and later exhumation through one or more surface processes, and (c) spatially concentrated aeolian erosion and wind energetics on well‐lithified basaltic substrates. To test the third hypothesis, we used the Mars Regional Atmospheric Modeling System to calculate wind erosive strength at 10 regions throughout the Martian highlands and compared it to their thermophysical properties by using thermal infrared data derived from the Thermal Emission Spectrometer to understand the effect that Amazonian mesoscale wind patterns may have on the exposure of bedrock. We also investigated the effect of planet obliquity, Ls of perihelion, and atmospheric mean pressure on wind erosion potential. We found no evidence for increased aeolian activity over bedrock‐containing regions relative to surrounding terrains, including at the mafic floor unit at Jezero crater (Máaz formation), supporting the first or second hypotheses for these regions.

## Key Points

- There is no observed relationship between simulated Late Amazonian wind patterns and the spatial bedrock exposures
- Flat‐lying surfaces in craters have lower wind erosion potential (WEP) values than intercrater plains. Jezero crater floor had the lowest values
- Increased atmospheric pressure had the strongest effect on calculated WEP values

## 1 Introduction

**Figure 1:**
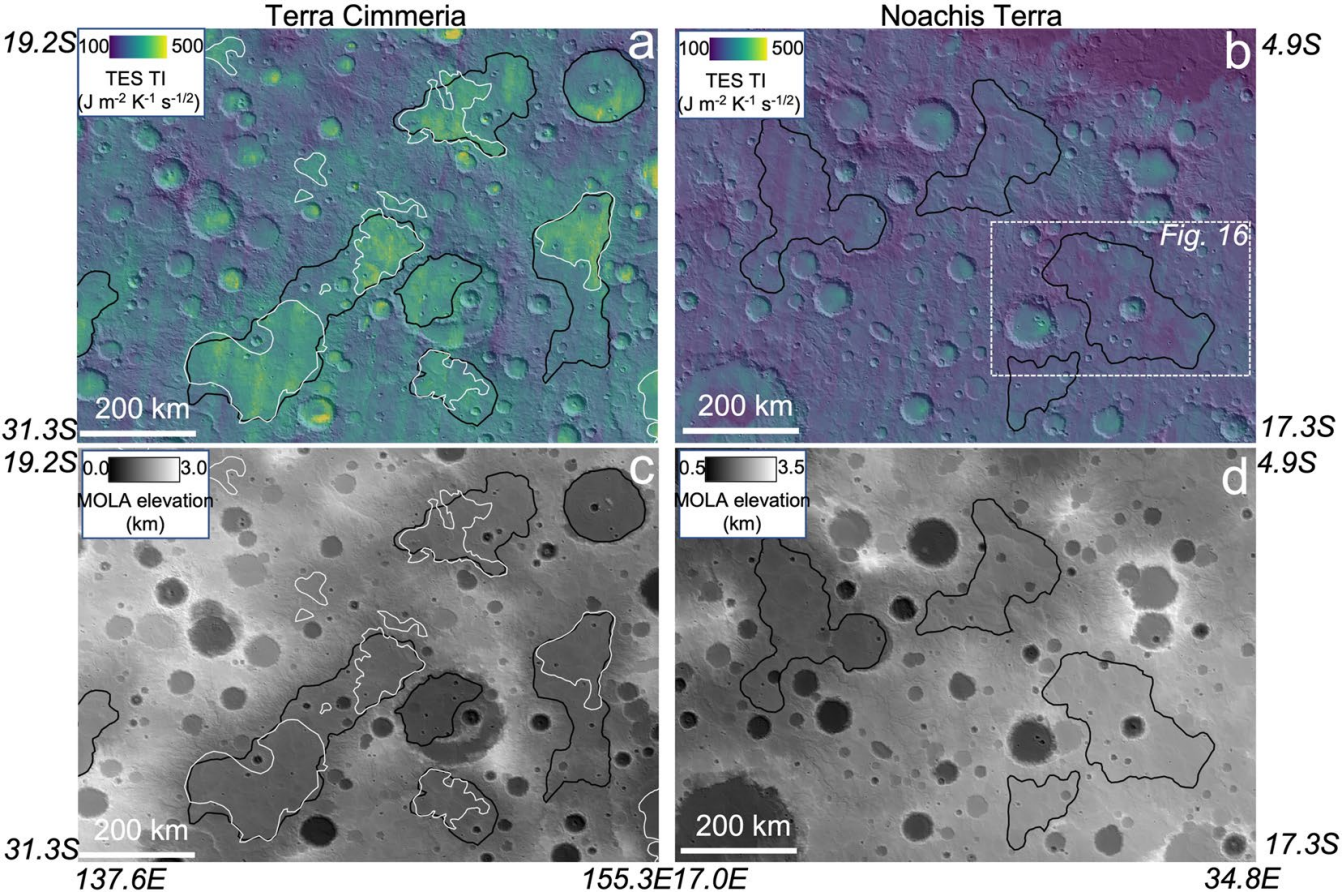
Examples of intercrater plain bedrock exposures mapped by Cowart et al. (2019) (white lines) and lNh units mapped by Tanaka et al. (2014) (black lines), for two regions. (a, b) Thermal Emission Spectrometer (TES) binned thermal inertia (Putzig et al., 2005) draped over Mars Orbiter Laser Altimeter (MOLA) shaded relief. (c, d) MOLA elevation draped over MOLA shaded relief. lNh units are commonly associated with relative topographic lows. Intercrater plain bedrock units are commonly found within lNh units but also in other Noachian and Hesperian units. The area shown in Noachis Terra (b, d) contains lNh units of similar size and planform shape to Terra Cimmeria but lacks bedrock.

Lower‐latitude Martian surface materials consist of a mixture of dust, sand, rocks and intact bedrock, all of which are now observable from orbit, and which are spatially variable across the surface (e.g., Christensen & Moore, 1992; Edwards et al., 2008). The recognition of intact bedrock at the surface first came from the Mars global surveyor (MGS) Mars Orbiting Camera which had high spatial resolution visible wavelength imaging (MOC, nominal spatial sampling of 1.4 m/pixel) (Malin et al., 1998) and from the Mars Odyssey Thermal Emission Imaging System (THEMIS) thermal infrared camera which has high resolution thermal wavelength imaging (100 m/pixel) (Christensen et al., 2004). Searches for bedrock in the Martian highlands have used THEMIS or MGS Thermal Emission Spectrometer (TES) temperature‐derived thermal inertia (TI) estimates to identify consolidated surface materials; from those efforts, it was found that vast (>200 km^2^), flat‐lying exposures of bedrock are found in many intercrater plains regions and filled craters (e.g., Cowart et al., 2019; Edwards et al., 2009; Rogers & Bandfield, 2009). These exposures were defined using a TES TI threshold of 350 J m^−2^K^−1^s^−½^ and commonly have full or partial spatial correspondence (Cowart et al., 2019) with the “Late Noachian highland” (lNh) units mapped by Tanaka et al. (2014) which are commonly found in broad, relatively flat topographic lows (e.g., Irwin et al., 2013) (Figure 1). In addition to the frequent association with Late Noachian‐aged units (Rogers & Nazarian, 2013), bedrock exposures exhibit degraded appearances, and in some places are superposed by Hesperian‐aged units (Rogers et al., 2018), all of which suggest that many are pre‐Hesperian and thus serve as records of Noachian resurfacing mechanisms. Some bedrock units, particularly in Terra Cimmeria, are large enough for crater counts using 1 km diameter craters, and exhibit Hesperian crater retention ages (Rogers et al., 2020). However, many are too small to obtain accurate emplacement ages from crater size frequency distributions and lack clear stratigraphic relationships with Hesperian materials, preventing a clear age determination (Rogers et al., 2018).

Since first discovery, these bedrock exposures have undergone multiple reinterpretations, in terms of their petrogenetic origin(s). They were originally interpreted as flood basalts (Ody et al., 2012; Rogers & Bandfield, 2009; Rogers & Fergason, 2011; Rogers & Nazarian, 2013), based on observations that the units exhibit olivine enrichments compared to surrounding lower TI materials, and lack both alteration minerals and morphologies (e.g., layering) associated with sedimentary deposits. However, more recent work by Rogers et al. (2018) supported the idea that some of these units may instead represent mechanically weak clastic deposits; their observations are summarized here. Bedrock exposures generally do not retain small craters compared to surrounding materials, and exhibit yardangs or other morphologies suggestive of friable material (Rogers et al., 2018). Also, in the few locations where Noachian bedrock units are directly subjacent to Hesperian volcanic units, the younger, Hesperian plains exhibit thicker regolith than the units they overlie (Rogers et al., 2018). One way to arrive at this present‐day configuration of thicker regolith on younger units is if there are differences in mechanical properties between the two units. Estimates of Martian cratering rates predict that a layer of regolith 10–100 m thick should exist on Noachian crustal materials due to impact gardening (Hartmann et al., 2001). Rogers et al. (2018) postulated that the lack of regolith on Noachian‐aged units was due to the material properties of the bedrock; namely that these are friable materials prone to deflation, thereby preventing regolith buildup.

However, bedrock friability may not fully explain the present‐day spatial distribution of rock exposure. For example, at Jezero crater, which is the landing site for the Mars 2020 rover mission, much of the crater floor material consists of a dark floor unit that exhibits mafic spectral features in orbital infrared data sets (the “mafic floor unit,” or “MFU,” Goudge et al., 2015) and retains craters of ∼100–300 m diameter (Warner et al., 2020). The MFU unit also exhibits variability in sediment cover, TI and apparent roughness (Ahern et al., 2021; Stack et al., 2020; Warner et al., 2020) and has been subdivided into “crater floor fractured rough” and “crater floor fractured smooth” units (Stack et al., 2020). Small craters within the MFU have well‐preserved, rocky rims, suggesting it is a competent material, consistent with crystalline igneous rock (Warner et al., 2020); in situ micro‐imaging observations from the Perseverance rover show interlocking, coarse crystals further suggesting a competent igneous rock is present within the MFU (Schmidt et al., 2022; Udry et al., 2022). The lack of a thick regolith at the MFU (particularly within the “crater floor fractured rough” subunit) compared to other known lava surfaces at the InSight (Warner et al., 2022) and Gusev crater landing sites (Golombek et al., 2006; Grant et al., 2006), led Warner et al. (2020) to suggest that perhaps the crater floor materials have experienced more vigorous surface processes than other known lava units or that the MFU was protected from small cratering by an overlying unit that was later removed. They also suggested that the TI and roughness differences across the floor may be due to enhanced stripping of regolith within the rough subunit of the MFU. Further in‐situ investigation and nominal sample return will resolve the unit lithology, but the process by which sediment was removed and the potential role of wind activity are still not well understood.

Similarly, some lNh bedrock exposures identified in Terra Cimmeria show higher crater retention and less morphologic evidence of deflation compared to other bedrock exposures, and also exhibit morphologies suggesting effusive lava origins (Cowart et al., 2019). This suggests that there may be other potential explanations for Noachian and Hesperian bedrock exposure, such as long‐term protection by overlying friable deposits followed by more recent exposure, or perhaps more vigorous aeolian erosion that strip regolith of fines, leading to higher TI.

A related observation is that some Noachis Terra lNh map units with similar surface morphologies and geologic settings to those in Terra Cimmeria *lack* bedrock exposures (Figure 1b), raising questions about whether they differ in material properties and/or depositional origin from those that do contain bedrock, or whether aeolian surface processes have been cumulatively weaker over these regions, preventing removal of comminuted fines. Disentangling the effects of primary depositional origin from surface modification via contemporary processes is important for deciphering the geologic record preserved in lNh units.

Understanding the relative roles of aeolian activity and mechanical properties in producing and maintaining bedrock exposure is key to better understanding bedrock origins. A fundamental unknown regarding these questions is how wind erosive strength varies on both regional and local scales. In this work, we test the hypothesis that spatial variability in wind strength is the primary control on present day bedrock exposure by using atmospheric modeling to assess whether regions with bedrock units have preferential, spatially‐concentrated wind activity compared to regions that lack bedrock units. We modeled wind activity over regions that contain lNh intercrater plains, intracrater plains, and bedrock exposures, and compare those values to Hesperian‐aged lava plains (Tanaka et al., 2014) that exhibit TI values consistent with extensive impact‐generated regolith, to help address some of the questions described above.

## 2 Study Regions

To understand the influence of wind patterns on bedrock exposure, atmospheric modeling must be at a sufficient spatial resolution to resolve the bedrock regions. Bedrock exposures mapped by Cowart et al. (2019) exceed 225 km^2^ in area, thus approximately 10 km/model element is the minimum scale necessary to resolve these surfaces. Such high spatial resolution prevents a global scale study and thus necessitates application to a few key areas that capture major surface units of interest described below.

**Figure 2:**
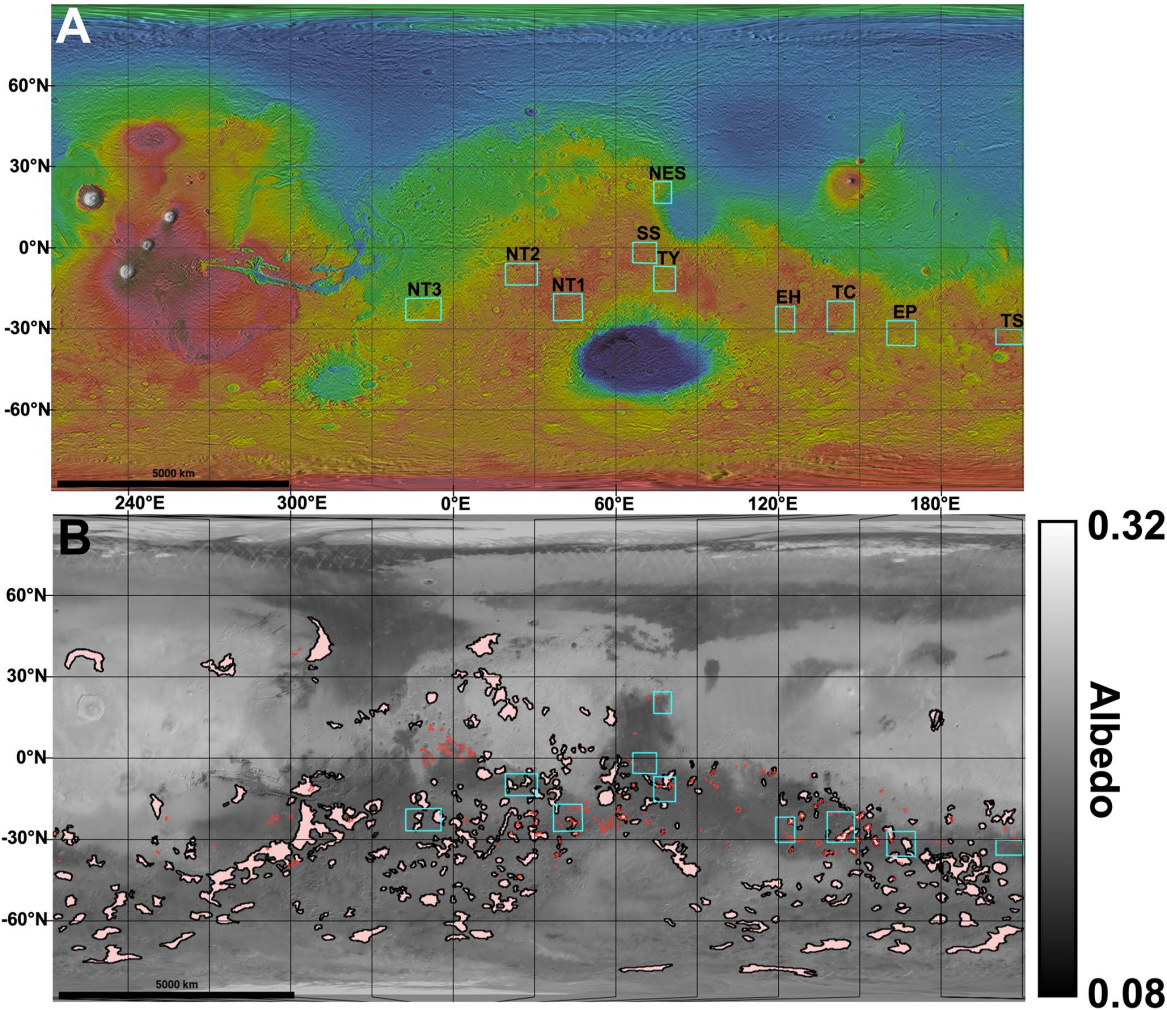
(a) Thermal Emission Imaging System/Context Camera/Mars Orbiter Laser Altimeter (MOLA) topographic mosaic of Mars. Light blue boxes indicate study regions. Letters indicate abbreviated location names (Table 1). (b) Thermal Emission Spectrometer albedo draped over MOLA shaded relief of Mars. Red outlines correspond to locations that contain bedrock exposures (Cowart et al., 2019). Pink polygons correspond to late Noachian highland units (lNh, Tanaka et al., 2014).

We selected 10 locations that are distributed throughout the highlands (Figure 2). All locations have Noachian‐aged units as per Tanaka et al. (2014) but only some contain late Noachian highland units (lNh) (Table 1). Three locations contain Hesperian‐aged volcanic units (Tanaka et al., 2014), and most contain bedrock exposures (Table 1). Regions without bedrock exposures were included for comparison. Detailed maps of bedrock distribution (defined by Cowart et al. (2019)), surface unit distribution (defined by Tanaka et al. (2014)), and TI (from Putzig et al., 2005) of each region are given in Figures S1–S10 in Supporting Information S1. Variability in elevation was also a factor when choosing these locations (Table 1). Bedrock exposures were taken from Cowart et al. (2019), who used a combination of a TES TI threshold of 350 J m^−2^K^−1^s^−½^ and elevated THEMIS nighttime radiance values to define the boundaries of bedrock exposures greater than 225 km^2^ in area (see Cowart et al., 2019 for details). Region “NES” includes Jezero crater (Goudge et al., 2015) and the MFU found within.

**Table 1:** Units Present in Our Chosen Study Locations

Location in Figure 1	Location name	lNh	Noachian bedrock	Hesperian regolith‐covered lava plains	Mean regional elevation (m)
NT1	Noachis Terra 1	Yes	Yes	No	1957
NT2	Noachis Terra 2	Yes	No	No	1853
NT3	Noachis Terra 3	Yes	No	No	−285
SS	South Syrtis Major Planum	No	Yes^a^	Yes	1858
TY	Tyrrhena Terra	Yes	Yes	No	1277
NES	Northeast Syrtis Major Planum^b^	No	Yes^a^	No	−1300
EH	East Hesperia Planum	Yes	Yes	Yes	1594
TC	Terra Cimmeria	Yes	Yes	No	1713
EP	Eridania Planitia	Yes	Yes	No	1071
TS	Terra Sirenum	No	Yes^a^	Yes	1660

^a^ Minimal amount of this unit found in the region, See mapped units in in Figures S1–S10 in Supporting Information S1 for each region.

^b^ Region that contains MFU.

## 3 Methods

A mesoscale atmospheric model was used to calculate and parameterize wind erosion potential (WEP) over the regions of interest (Section 3.1). To relate WEP to material properties, we used TI products derived from TES (Section 3.2). Modeled WEP values were then sampled from surface units of varying age and material properties (Section 3.3) in order to address the objectives listed in Section 1 and enable both inter‐ and intra‐regional comparisons of WEP.

### 3.1 Mesoscale Atmospheric Modeling

#### 3.1.1 The Mars Regional Atmospheric Modelling System (MRAMS)

For each of our regions of interest, the Mars Regional Atmospheric Modeling System (MRAMS, Rafkin & Michaels, 2019; Rafkin et al., 2001) was used to calculate the detailed local Late‐Amazonian atmospheric environment for a diverse set of past conditions. MRAMS simulates the non‐hydrostatic and fully compressible Martian atmosphere over a given regional scope, using realistic topography, surface characteristics, and radiative transfer (Rafkin & Michaels, 2019; Rafkin et al., 2001). As a regional model, it uses an initial state and time‐dependent boundary conditions obtained from prior Mars Global Circulation Model (MGCM) output—in this case, the NASA Ames MGCM (Haberle et al., 1993, 2019).

Aeolian erosive strength of mesoscale winds was parameterized by calculating WEP at each MRAMS horizontal grid point and output timestep. WEP flux is the predicted sand saltation mass flux based on localized surface shear velocity values (Equation 2.34 in Kok et al., 2012), assuming unlimited sand supply and unfettered sand mobility. It has units of (kg m^−1^ s^−1^), rather than the more familiar per‐area flux units, since it is integrated vertically over the depth of atmosphere that the saltating particles are moving through. Greater WEP flux values thus indicate a higher wind erosive strength, as per:

(1) $${\text{WEP}}_{f}=C\frac{\rho_{a}}{g}\;u_{{*}_{\text{it}}}\left(u_{*}^{2}-u_{{*}_{\text{it}}}^{2}\right)$$

Where *C = gL*_msh_/$u_{{*}_{\mathrm{it}}}$Δ*v*_*ivl*_ is a unitless scaling factor that includes the effect of the particles' saltation hop length (m) (*L*_msh_ ≈ 1 m for Mars; Kok et al., 2012) and the average difference between their impact and liftoff speeds (Δ*v*_*ivl*_ ≈ 1 m s^−1^; Kok et al., 2012), *ρₐ* is the density of Martian air (kg/m^3^), g is Martian gravity (m/s^2^), *u*_∗_ is the surface shear velocity (conceptually, the speed at which the air pushes against the taller surface roughness elements) (m/s), and $u_{{*}_{\mathrm{it}}}$ is the impact threshold shear velocity (i.e., the shear velocity at which saltation can be sustained *after being initiated*) (m/s). After Kok (2010), $u_{{*}_{\mathrm{it}}}$ is taken to be 10% of the instantaneous fluid threshold shear velocity (calculated via the technique of Greeley & Marshall (1985)). To determine whether saltation *initiation* was likely at a given MRAMS timestep and grid point, it is important to note that numerical models like MRAMS compute the spatial‐mean (Reynolds‐averaged) wind within each three‐dimensional grid cell at any given time, but in reality, there are also smaller‐scale winds that cannot be resolved by finite model grid spacing (e.g., turbulent gusts, Bridges et al., 2012). Using the technique described in Stillman et al. (2021), we removed some nominally non‐zero model output WEP values at times and locations where sub‐grid‐scale turbulent wind gusts able to initiate saltation were not likely to exist.

Mesoscale wind patterns are dependent on orbital and axial parameters of Mars which are known to have varied in the past. Thus, our models were run with multiple values of axial obliquity, *L*_*s*_ of perihelion, and global‐average atmospheric pressure (Table 2). Values of *L*_*s*_ of perihelion (*ϖ*) were set to values of 251°, 71°, or 90°. The first two values account for the axial precession variability of Mars that occurs at a 10⁵ Earth‐year cycle (Parry Rubincam, 1999), both at the current value (251°) and at 180° from that. The *ϖ* value of 90° was used to maximize insolation during the northern hemisphere summer solstice for a very low obliquity (14.7°) case. With regard to obliquity (***ε***), Laskar et al. (2004) suggested that the obliquity variability of Mars has followed a Gaussian distribution for the past 250 million years and anything before has unquantifiable chaotic behavior (Laskar et al., 2004). In this work we used four different sample obliquity values, based on the calculations of Laskar et al. (2004): Mars' current obliquity (25.19°), Mars' average obliquity (34.64°), and approximately one standard deviation about the mean (54.61°, 14.71°) (Laskar et al., 2004). Lastly, we varied the global mean atmospheric surface pressure (*p*_*sf*_) of the atmosphere using Mars' current average value (∼7 mbar, e.g., Harri et al., 2014) and double the current value (14 mbar). The doubled value is based on prior work that showed that the south polar region of Mars has a sequestered volume of CO_2_ ice sufficient to double Mars' current atmospheric mass if it were released due to obliquity variations (Bierson et al., 2016; Jakosky & Edwards, 2018; Phillips et al., 2011). With the different parameters mentioned above, we arrive at 12 different simulated cases, plus one “Case 0a,” described below (Table 2). It is important to note that the global mean atmospheric surface pressure parameter is only used to initialize the atmospheric mass inventory of the MGCM—the full daily and annual atmospheric pressure cycles are simulated by the MGCM (and later conferred to the MRAMS runs). The limited knowledge of orbital and atmospheric conditions prior to ∼250 Mya (Laskar et al., 2004) limits our assessments and interpretations to Late‐Amazonian wind activity only.

**Table 2:** Simulation Cases for Which MGCM and MRAMS Modeling Were Performed, Intended to Sample Different Atmospheric, Axial, and Orbital Parameter Configurations Experienced by Mars in the Relatively Recent Past

Calculated cases	Obliquity (*𝜺*)	*L* *_s_* of Perihelion (*𝝕*)	Mean global atmospheric pressure (𝑝_*sf*_)
Case 0a	25.19°	251°	7 mbar
Case 1a	25.19°	251°	7 mbar
Case 1b	25.19°	251°	14 mbar
Case 2a	34.64°	251°	7 mbar
Case 2b	34.64°	251°	14 mbar
Case 3a	34.64°	71°	7 mbar
Case 3b	34.64°	71°	14 mbar
Case 4a	54.61°	251°	7 mbar
Case 4b	54.61°	251°	14 mbar
Case 5a	54.61°	71°	7 mbar
Case 5b	54.61°	71°	14 mbar
Case 6a	14.71°	90°	7 mbar
Case 6b	14.71°	90°	14 mbar

*Note*. Case 0a (in red) is the nominal current Mars configuration, and has a more complex atmospheric dust loading prescription than the other cases.

Case 0a is the same as Case 1a (Table 2), except that it uses the full spatially‐ and temporally‐variable atmospheric dust loading climatology (Mars Year 24) of Montabone et al. (2015), which is a function of latitude, longitude, and *L*_*s*_. Since the details of the annual dust cycle in prior Late‐Amazonian eras are not known, but the topography and other surface properties (e.g., TI and albedo) can be assumed to be very similar to the present, all other (paleo‐Mars) cases use a muted form of the Mars Year 24 dust climatology, where the maximum dust opacity is not permitted to exceed the equatorial zonally‐averaged atmospheric dust opacity value at *L*_*s*_ = 180°. This muted form is intended to satisfactorily reflect the robust high‐latitude annual atmospheric dust loading patterns (i.e., much less dust over the winter high‐latitudes, more dust over the summer ones), while suppressing present‐day maxima that are related to the current *L*_*s*_ of perihelion. The time‐ and location‐dependent vertical distribution of the dust is dynamically parameterized taking into account the daily insolation and dust opacity (tuned to contemporary orbital observations), and so adapts reasonably well to these paleo‐Mars model configurations. Even though such a prescription is not ideal, it allows comparison of the overall wind (and thus erosive potential) changes that axial/orbital variations induce.

For each case, daily WEP (kg m^−1^) for four seasonal bins (centered at *L*_*s*_ 10°, 100°, 190°, and 280°, Allison & McEwen, 2000) was calculated by time integrating WEP flux. To reduce the amount of data analyzed, annual WEP values (kg m^−1^) were computed using a weighted sum of the four *L*_*s*_ bins' WEP values, with the weights based on the number of sols in each 90°‐wide seasonal bin (non‐constant due to Mars' significant orbital eccentricity and the varying *L*_*s*_ of perihelion).

**Figure 3:**
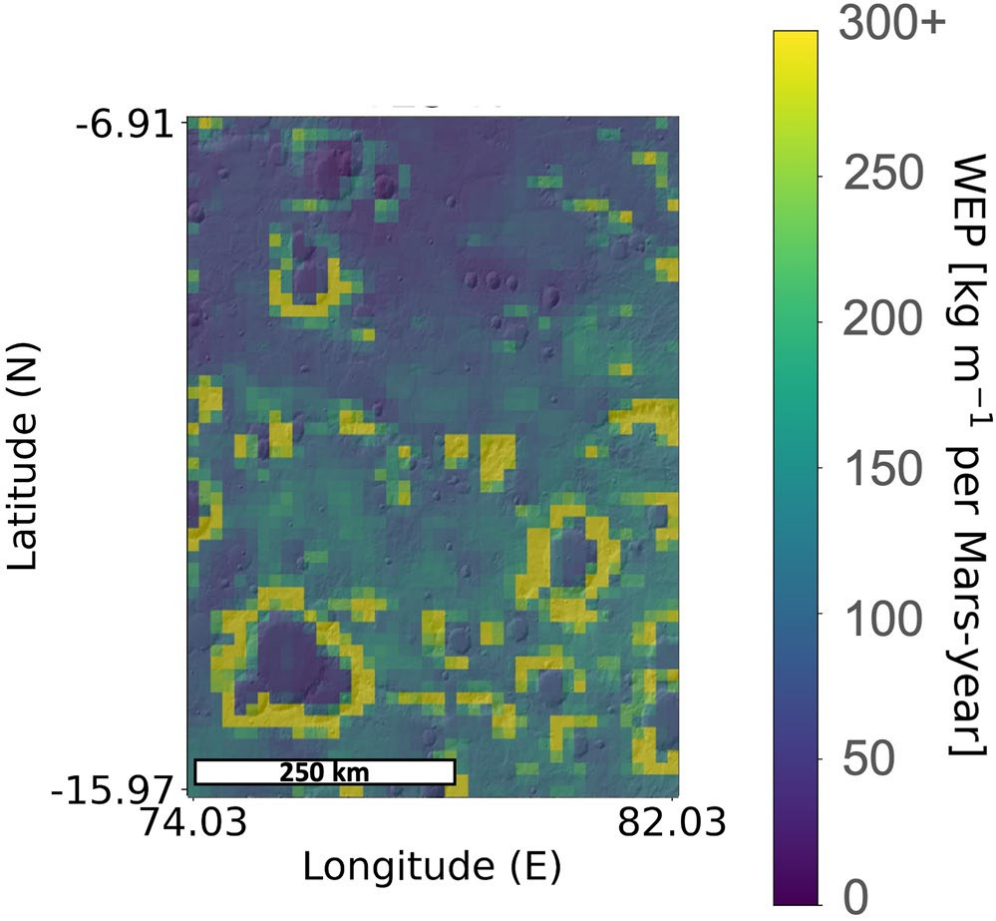
Spatial distribution of Mars Regional Atmospheric Modeling System annual Wind erosion potential (kg m^−1^ per Mars‐year) values at Tyrrhena Terra for case 1a.

Wind erosion potential values were calculated at a sampling of ∼8 km/pixel for all regions. Example output can be seen in Figure 3 which shows the spatial distribution of annual WEP at Tyrrhena Terra for case 1a. WEP maps for the remaining regions are found in Figures S1–S10 in Supporting Information S1. To resolve potential annual WEP variations across the Jezero MFU, WEP was also calculated with a finer spatial sampling of ∼2.7 km/pixel for the MFU.

#### 3.1.2 WEP Assumptions and Model Validation

Our WEP calculations assume that there is an infinite supply of mobile sand to participate in saltation at each region. This assumption is likely invalid in many places, given that orbital and rover‐based observations have shown great spatial variability in sediment supply and mobility (e.g., due to surface armoring and cohesion). This is why WEP is only a metric for the potential magnitude of aeolian erosion if it should/can occur, and is not itself sufficient to determine whether erosion is occurring.

Several studies have compared surface or geomorphic observations with MRAMS model predictions and serve as a means for model validation. Comparisons of meteorological data (e.g., wind speeds/direction, air pressure, air temperature) at the Ares Vallis (Mars Pathfinder) and Gale crater (Mars Science Laboratory) landing sites have been shown to compare well with MRAMS predictions (Pla‐Garcia et al., 2016; Rafkin et al., 2001). Calculated shear stresses and wind directions for the Mars Pathfinder landing site were found to be consistent with crater rim erosional morphologies at the site (Kuzmin et al., 2001), and modeled wind direction data at Gusev crater was in good agreement with the direction and dynamics of a dust devil observed by the Mars Exploration Rover cameras (Greeley et al., 2006). Several orbital‐based studies demonstrated good agreement between MRAMS predictions and aeolian features such as dunes, wind streaks and erosional patterns observed at various locations with remotely sensed data (Chojnacki et al., 2011; Greeley & Thompson, 2003; Hobbs et al., 2010; Silvestro et al., 2013). For example, a study found that MRAMS modeled wind directions were consistent with observed barchan dune migration directions from HiRISE imaging (Fenton et al., 2014). MRAMS‐derived meteorological data for the Phoenix Lander mission were found to be consistent with meteorological parameters derived from MGS radio science and the TES instrument over the Phoenix site (Michaels & Rafkin, 2008).

### 3.2 Thermal Inertia

We used TI (TI, [J m^−2^ K^−1^ s^−1/2^ or tiu]) as a proxy for material properties (e.g., bedrock exposure and regolith cover). Thermal inertia is a thermophysical property that describes the capability of any given material to resist changes in temperature (e.g., Kieffer et al., 1977). This property is affected by the material's bulk density (*ρ*, [kg/m^3^]), specific heat capacity (*c*, [J/kg K]), and thermal conductivity (*k*, [J/s m K]) as seen in the following equation:

(2) $$\text{TI}\;=\;\sqrt{k\rho c}$$

Materials that are more indurated such as bedrock tend to have higher TI compared to unconsolidated materials such as sand or sediment (Christensen & Moore, 1992; Kieffer et al., 1977). Within unconsolidated materials, TI increases with grain size (e.g., Presley & Christensen, 1997).

Putzig and Mellon (2007) created a quantitative global TI map using MGS TES nighttime temperature observations (Christensen et al., 2001). Their map was produced at 20 pixels per degree (ppd) resulting in ∼3 km/pixel resolution at the equator, which is similar to the spatial sampling of our MRAMS model output (Section 3.1). This map contains artifacts in the Terra Cimmeria region, where certain orbit tracks exhibit significantly higher derived TI values. The artifacts are possibly due to seasonal or episodic atmospheric conditions that were not well treated in the complex TI calculations. Thus, for that region we instead used an older TES TI map that has the same spatial resolution of 20 ppd (Putzig et al., 2005). Outside of the elevated values associated with orbit tracks, the older and newer TI maps showed insignificant differences in this region. The MGS TES investigation used the International Astronomical Union (IAU) 1994 (Davies et al., 1995) coordinate system, which results in a longitudinal offset from other data sets that use the IAU 2000 (Seidelmann et al., 2002) coordinate system (e.g., Rogers et al., 2005); we corrected the TES data for the longitudinal offset to match all other data sets used in this study.

Given that MRAMS WEP and TES TI data had slightly different resolutions, it was necessary to bin the TES data to match MRAMS resolution. We did this by creating a grid of bins with assigned latitude and longitude ranges. We took the average of both MRAMS and TES datasets that existed within the latitude and longitude ranges for each bin matching the dimensions of the created grid. Both resulting datasets had the same geography and dimensions, allowing them to be better compared with each other. When binning the datasets, the means of both TI and WEP values at each bin may not be precisely representative of that region, but are close enough to be considered characteristic of each bin of ∼8 km/pixel.

### 3.3 Surface Unit Delineation

**Figure 4:**
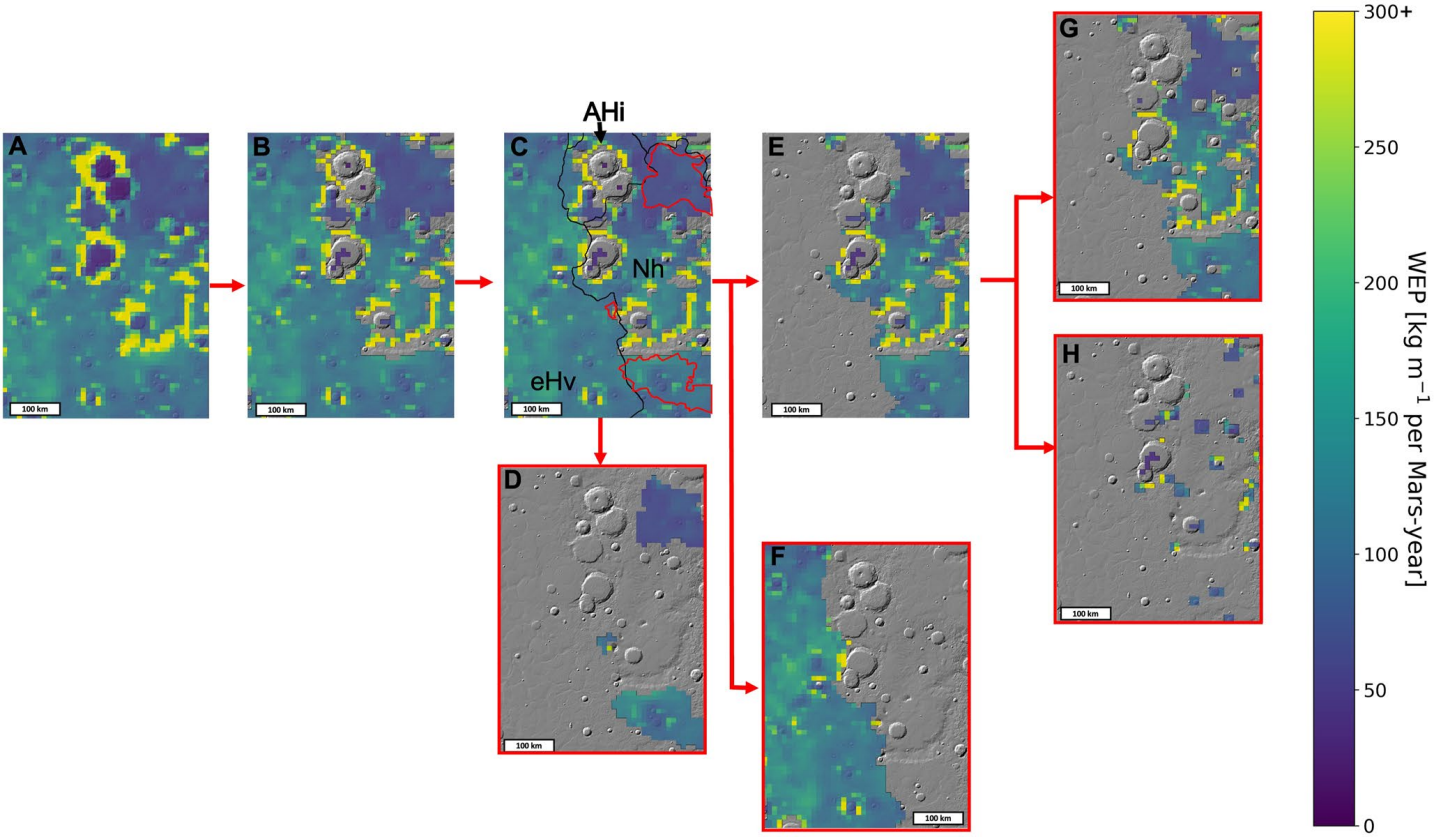
Example of algorithm for surface unit delineation and analysis using data from case 0a at East Hesperia Planum. Data used for further analysis outlined in red. (a) Annual Wind erosion potential (WEP) (kg m^−1^ per Mars‐year) data set in binned resolution after performing a weighted sum of seasonal daily WEP. (b) Highly‐sloped regions are eliminated to facilitate direct comparison between bedrock‐containing plains (generally flat) and other flat plains that lack bedrock. This was necessary because slope has a non‐trivial effect on wind activity and strength. (c) Delineation of units in region using Tanaka et al., 2014 units (black outlines), with red outlines indicating bedrock exposures (Cowart et al., 2019). (d) Bedrock outcrops extracted from low‐slope data using regions delineated in step (c) Separation and extraction of geologic units delineated in step led to (e) Noachian highland (Nh) unit and (f) early Hesperian volcano unit (eHv). We further subdivided the Nh units to analyze: (g) Noachian intercrater plains and (h) Noachian intracrater plains separately.

Major surface units were delineated to facilitate sampling of WEP and TI from flat‐lying units of similar age and/or material properties. Because all of the bedrock exposures are relatively flat‐lying at the resolution of our study, our first step was to mask out WEP and TI pixels located on sloped surfaces. This permits a more direct statistical comparison of WEP values from bedrock units to WEP from plains that do not contain bedrock. To do this, we first calculated the slope value of each bin using MGS Mars Orbiter Laser Altimeter (MOLA) elevation data with a spatial resolution of 463 m/pixel spatial resolution (Smith et al., 2001) as a baseline and a moving‐window slope calculation algorithm. Our slope threshold was set to 0.75° through an iterative approach to determine the highest value that effectively excluded large crater rims and scarps for all of our study regions (Figure 4b).

Slope‐thresholded WEP and TI values were further spatially classified using previously published surface units (Goudge et al., 2015; Tanaka et al., 2014) and bedrock (Cowart et al., 2019) maps, as well as geologic context described below (Figure 4c). WEP and TI values were extracted from locations that matched bedrock exposures using the map generated by Cowart et al., 2019 (Figure 4d). USGS geologic map #3292 (map scale of 1:20,000,000, Tanaka et al., 2014) was used to extract TI and WEP values from map units designated as Noachian highlands units (early, middle and late Noachian highland units [eNh, mNh, lNh] (Figure 4e) and Hesperian volcanic units (Hvu) (Figure 4f). For simplicity, we grouped all Noachian highland units into a single unit. We then subdivided the Noachian highland unit into Noachian intercrater plains (Figure 4g) and intracrater plains (surfaces contained within craters, Figure 4h). Thus, we extracted TI and WEP data for four different units for comparison: bedrock exposures, Hesperian lava plains, Noachian intercrater plains, and Noachian intracrater plains (outlined in red in Figure 4). We used the Pearson correlation coefficient (Freedman et al., 2007) to assess possible spatial relationships between TI and WEP. Note that our Hesperian lava plains, Noachian intercrater plains, and Noachian intracrater plains units are inclusive of the bedrock units described above; this was done to compare map units of interest that contain bedrock with those that do not. Last, we also sampled WEP values from the MFU at Jezero crater, using map boundaries from Goudge et al. (2015).

## 4 Results

**Figure 5:**
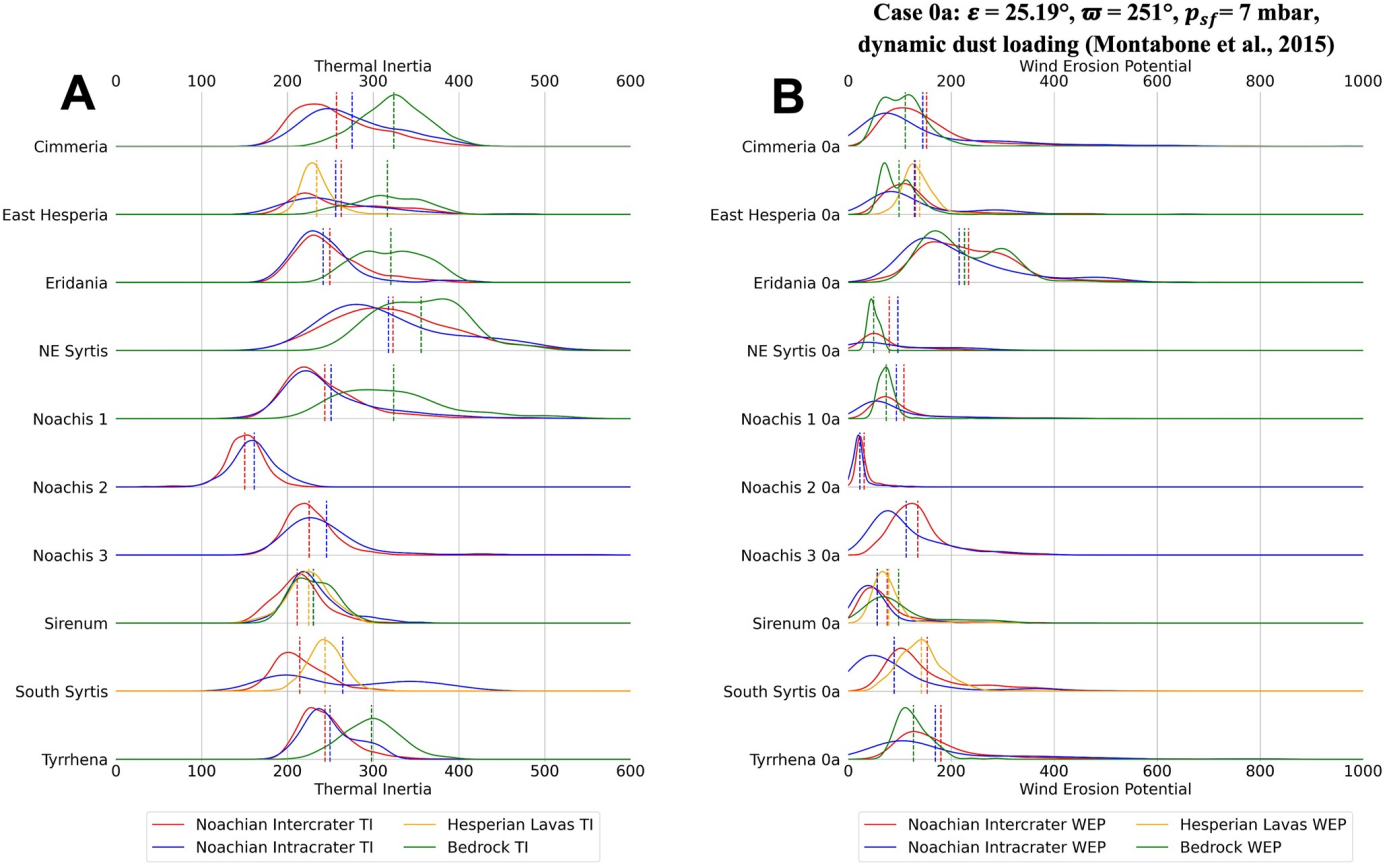
(a) Stacked distribution plots showing TES TI distributions of each location for all analyzed regions in this study. (b) Stacked distribution plots of Mars Regional Atmospheric Modeling System annual Wind erosion potential value distributions at each region for case 0a **(*ε* = 25.19°, ϖ = 251°,** *p*_*sf*_ **= 7 mbar, nominal atmospheric dust loading)**. Dashed lines indicate the means of distributions.

**Figure 6:**
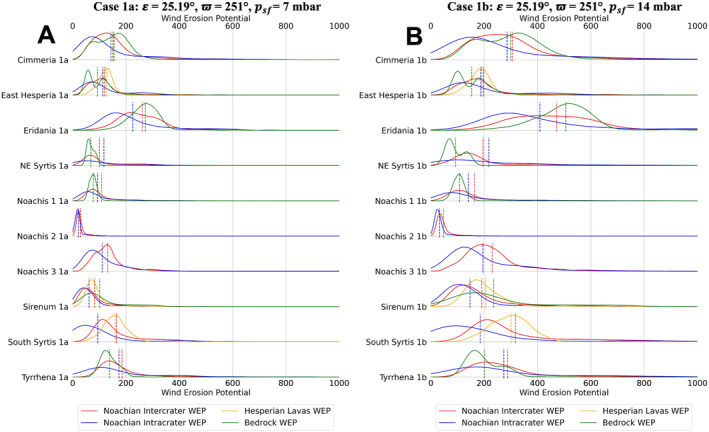
Stacked distribution plots for all analyzed locations in this study. Dashed lines indicate the means of distributions. (a) Mars Regional Atmospheric Modeling System (MRAMS) annual Wind erosion potential (WEP) value distributions at each region for case 1a **(*ε* = 25.19°, ϖ = 251°,** *p*_*sf*_ **= 7 mbar)** (b) MRAMS annual WEP value distributions at each region for case 1b **(*ε* = 25.19°, ϖ = 251°,** *p*_*sf*_ **= 14 mbar)**.

**Figure 7:**
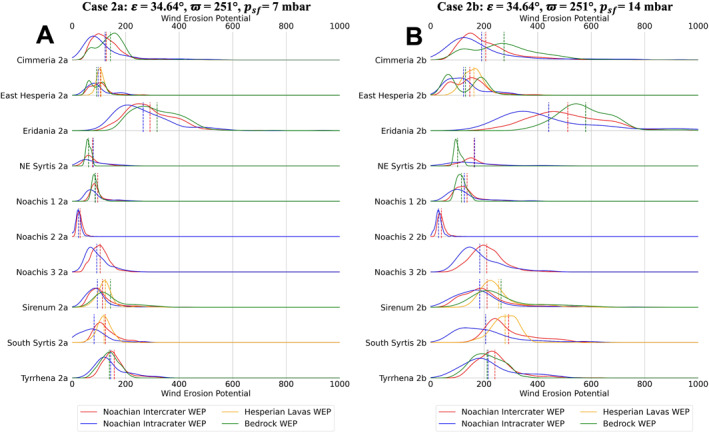
Stacked distribution plots for all analyzed locations in this study. Dashed lines indicate the means of distributions. (a) Mars Regional Atmospheric Modeling System (MRAMS) annual Wind erosion potential (WEP) value distributions at each region for case 2a **(*ε* = 34.64°, ϖ = 251°,** *p*_*sf*_ **= 7 mbar)** (b) MRAMS annual WEP value distributions at each region for case 2b **(*ε* = 34.64°, ϖ = 251°,** *p*_*sf*_ **= 14 mbar)**.

**Figure 8:**
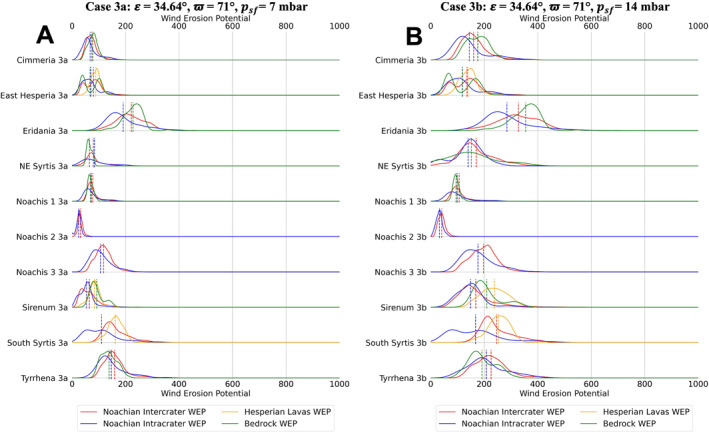
Stacked distribution plots for all analyzed locations in this study. Dashed lines indicate the means of distributions. (a) Mars Regional Atmospheric Modeling System (MRAMS) annual Wind erosion potential (WEP) value distributions at each region for case 3a **(*ε* = 34.64°, ϖ = 71°,** *p*_*sf*_ **= 7 mbar)** (b) MRAMS annual WEP value distributions at each region for case 3b **(*ε* = 34.64°, ϖ = 71°,** *p*_*sf*_ **= 14 mbar)**.

**Figure 9:**
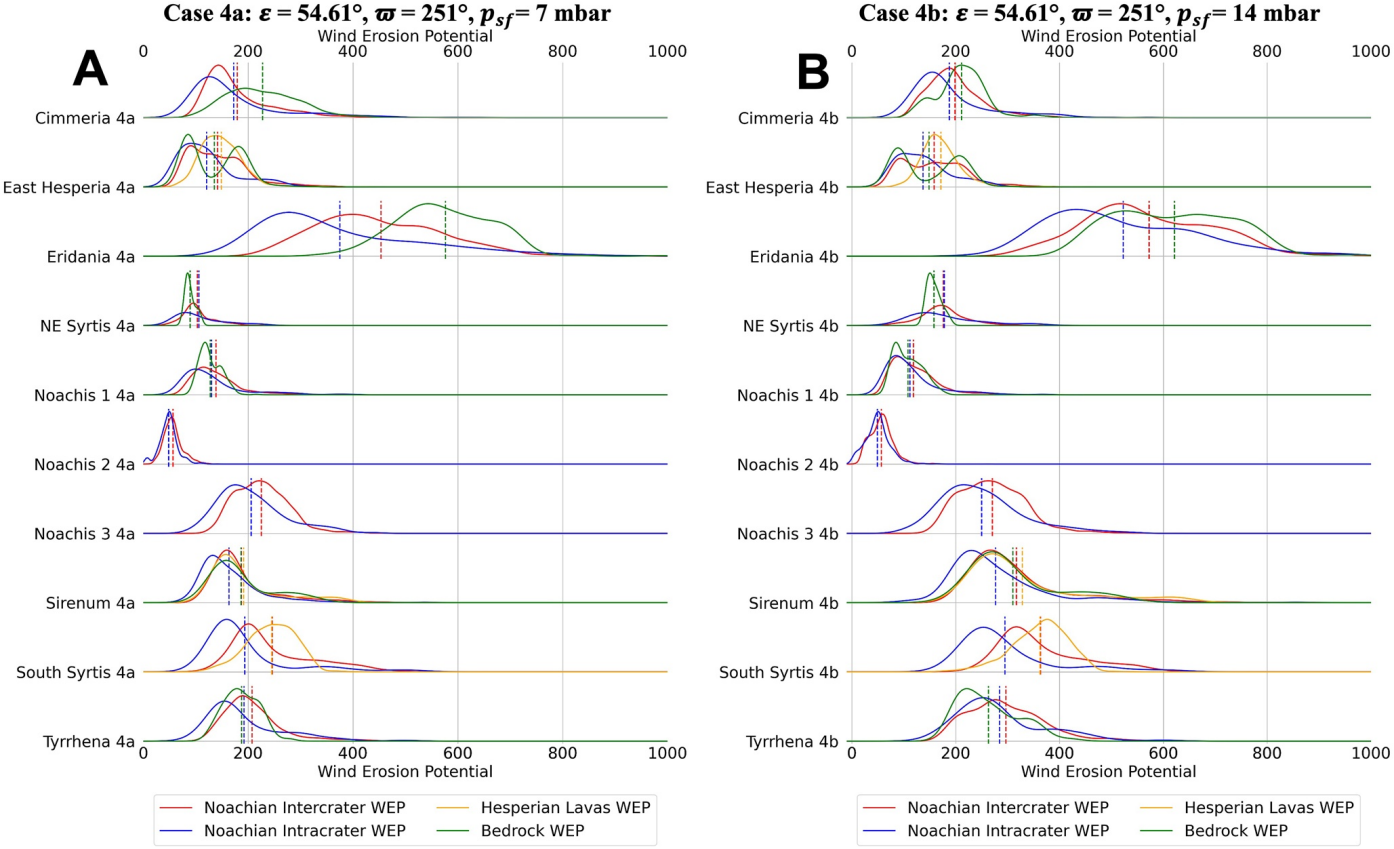
Stacked distribution plots for all analyzed locations in this study. Dashed lines indicate the means of distributions. (a) Mars Regional Atmospheric Modeling System (MRAMS) annual Wind erosion potential (WEP) value distributions at each region for case 4a (***ε*** = **54.61**°, **ϖ** = **251**°, *p*_*sf*_ = **7** **mbar**) (b) MRAMS annual WEP value distributions at each region for case 4b (***ε*** = **54.61**°, **ϖ** = **251**°, *p*_*sf*_ = **14** **mbar**).

**Figure 10:**
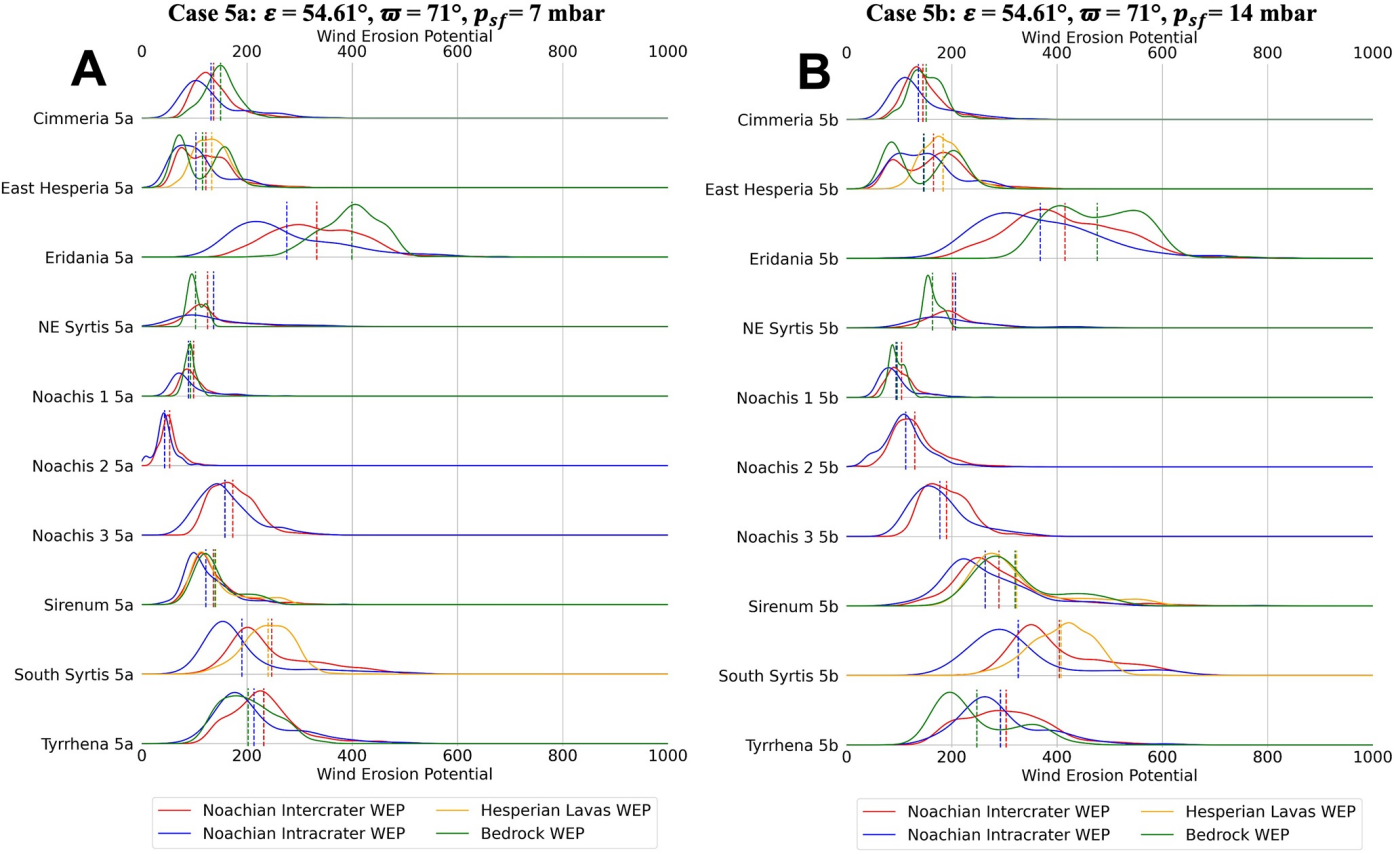
Stacked distribution plots for all analyzed locations in this study. Dashed lines indicate the means of distributions. (a) Mars Regional Atmospheric Modeling System (MRAMS) annual Wind erosion potential (WEP) value distributions at each region for case 5a (***ε*** = **54.61**°, **ϖ** = **71**°, *p*_*sf*_ = **7** **mbar**) (b) MRAMS annual WEP value distributions at each region for case 5b **(*ε*** = **54.61**°, **ϖ** = **71**°, *p*_*sf*_ = **14** **mbar**).

**Figure 11:**
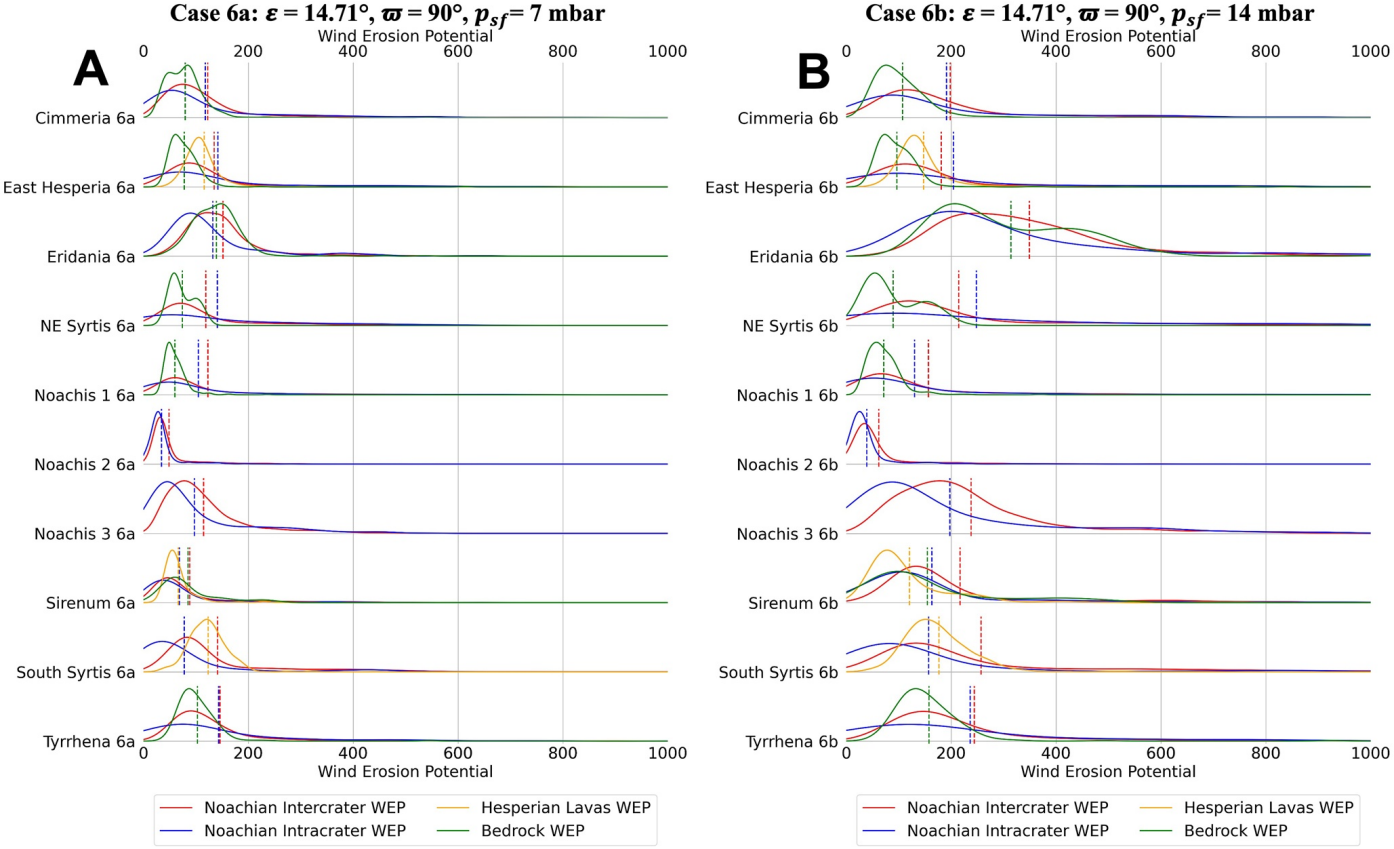
Stacked distribution plots for all analyzed locations in this study. Dashed lines indicate the means of distributions. (a) Mars Regional Atmospheric Modeling System (MRAMS) annual Wind erosion potential (WEP) value distributions at each region for case 6a **(*ε*** = **14.71**°, **ϖ** = **90**°, *p*_*sf*_ = **7** **mbar**) (b) MRAMS annual WEP value distributions at each region for case 6b (***ε*** = **14.71**°, **ϖ** = **90**°, *p*_*sf*_ = **14** **mbar**).

Distributions of TI and annual WEP for climate case 0a are shown for each unit type, for each of the 10 study regions, in Figure 5. The distributions were created by using a kernel density estimator on each data set. Figures 6, 7, 8, 9, 10, 11 show distributions for climate cases 1–6a and 1–6b, for each unit type within each of the 10 study regions. We did not generate histograms for the Jezero MFU due to small sample size (44 pixels). However, the MFU mean WEP values and distributions are shown in Figures 13 and 14.

### 4.1 TI Characteristics of Surface Unit Classes

At all locations the TI distributions and mean values for Noachian intercrater and intracrater plains tend to be similar, with mean values typically within ∼20 J m^−2^ K^−1^ s^−½^ of one another, and generally found between ∼210 and 275 J m^−2^ K^−1^ s^−½^ (Figure 5). These values are consistent with an effective particle size of ∼200–550 μm, using laboratory‐determined thermal conductivity values measured by Presley and Christensen (1997) and a volumetric heat capacity of 1 × 10^6^ J m^−3^ K, after Neugebauer et al. (1971), and are typical of Martian low‐albedo regions (Putzig et al., 2005). One exception is in Noachis 2, where plains TI values are ∼150 J m^−2^ K^−1^ s^−½^, consistent with effective grain size of ∼60 μm (e.g., Presley & Christensen, 1997). Bedrock units exhibit TI values that are typically ∼50–100 J m^−2^ K^−1^ s^−½^ higher than Noachian inter/intracrater plains and Hesperian volcanic units, with mean values typically greater than 300 J m^−2^ K^−1^ s^−½^. The higher TI values in bedrock units were expected because bedrock units were identified by Cowart et al. (2019) on the basis of elevated TES TI values. Thermal inertia values of 300 J m^−2^ K^−1^ s^−½^ correspond with an effective grain size of ∼900 μm if unconsolidated materials; however, high‐resolution imaging over the bedrock regions mapped by Cowart et al. typically shows intact rock exposures with varying textures and patchy sand cover. This suggests that the TES TI values represent subpixel mixtures of exposed rock and unconsolidated sands, but it is possible that these surfaces could also contain significant fractions of coarse sands and gravels. At South Syrtis Major Planum and Terra Sirenum, the TI values are higher in the Hesperian units than Noachian intercrater plains but lower than the intracrater plains. At East Hesperia Planum the inter/intracrater plains have higher TI than Hesperian lava plains (Figure 5).

### 4.2 Changes in Annual WEP With Climate State

**Figure 12:**
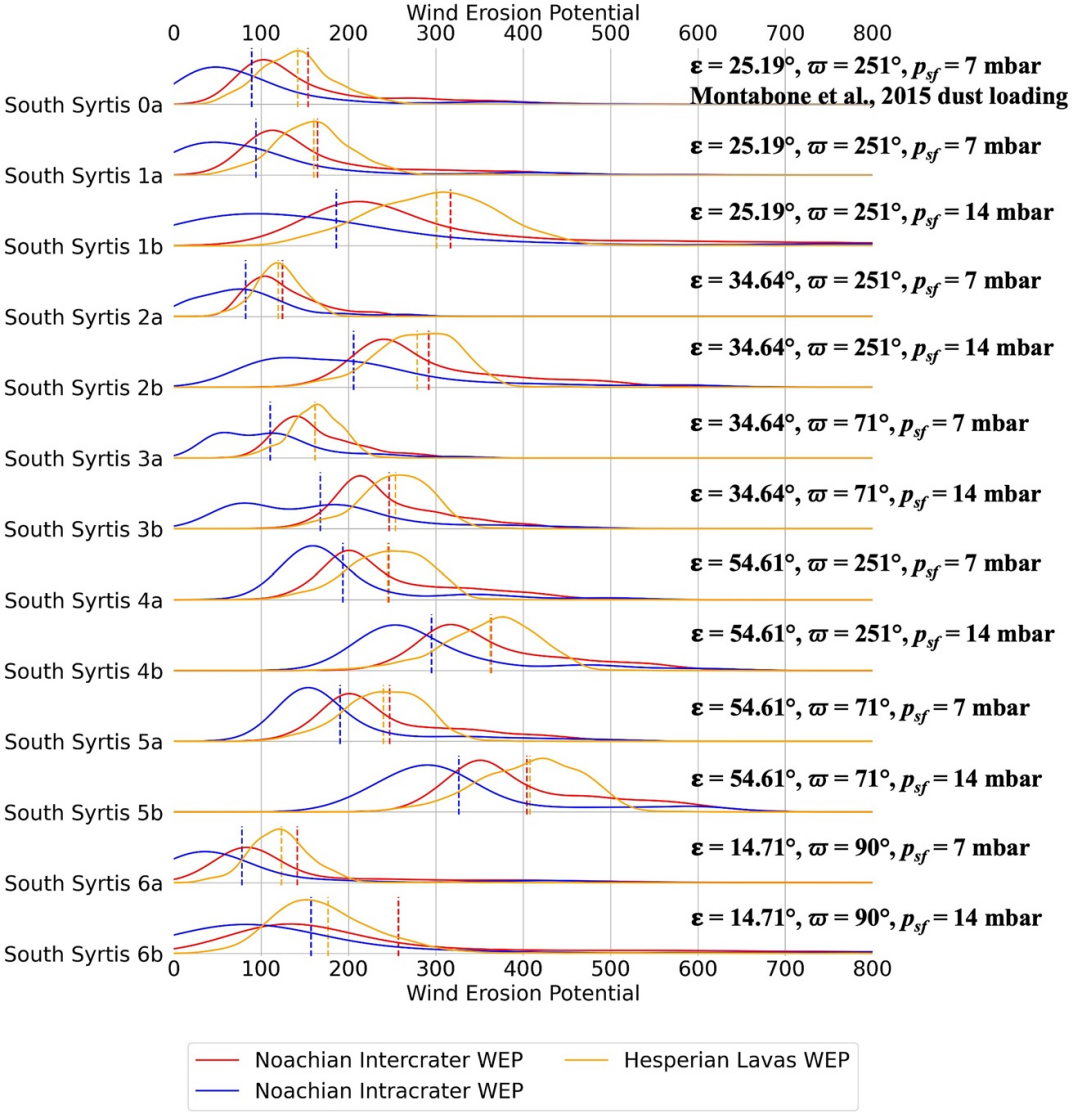
Stacked distribution plots of annual Wind erosion potential for all modeling cases for South Syrtis Major Planum. Dashed lines indicate the means of distributions.

**Figure 13:**
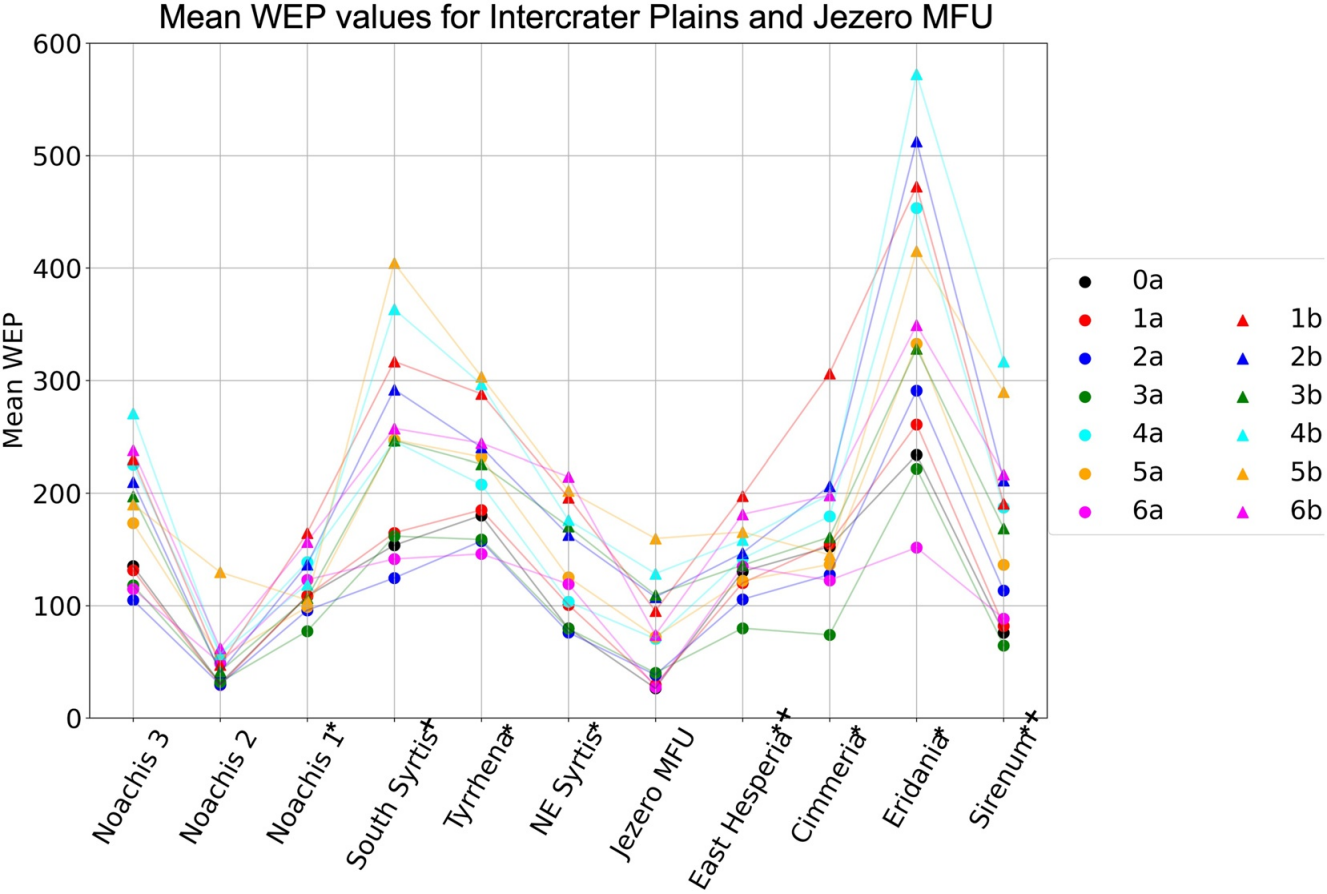
Scatter plot of mean annual Wind erosion potential (kg m^−1^ per Mars‐year) for Noachian intercrater plains at each region and the Jezero mafic floor unit, for each modeling case. Cases with the letter “a” are depicted as circles, while cases with the letter “b” are depicted as triangles. *Locations with bedrock exposures. **⁺**Locations with Hesperian lava plains.

To help visualize changes in annual WEP with the different modeling cases, we plot our data in two ways. Figure 12 shows the effect of each modeling case on WEP for individual surface units within one study region, South Syrtis. Figure 13 shows the effect of each modeling case on WEP for one type of surface unit (intercrater plains) for all regions. Changes in WEP were found to be generally the same from region to region, where modeling cases that yielded the greatest WEP for one region generally yielded highest WEP in other regions, with the exception of Noachis 2, which showed higher WEP from climate state 5b. Some differences in the magnitude of WEP changes were observed from region to region (Figure 13). For most of the discussion that follows in this section, the reader can refer to Figures 12 and 13 except where otherwise noted.

We compared Muted versus nominal dust loading (Case 1a vs. Case 0a) at each region and we found that the WEP distributions for Case 1a and Case 0a had similar shapes and mean values for all regions, with the exception of the bedrock surface unit in Eridania Planum, which showed a bimodal distribution in case 0a that was absent in case 1a (Figures 5b and 6a).

We assessed the effect of atmospheric pressure variations (“a” vs. “b” cases) at all locations, where we found doubling the global mean atmospheric surface pressure from 7 to 14 mbar (for MGCM initialization) resulted in an increase in WEP values and a broadening of the WEP distributions (Figure 12). Increasing the atmospheric pressure to 14 mbar led to an increase by a factor of 1.7 ± 0.1 in WEP values for all regions on average, making it the model input with the most impact when calculating erosive patterns in the region.

We also compared current Martian_*s*_ obliquity (25.19°) to a less‐tilted Mars (14.71°), and we found there was no significant difference in WEP values (e.g., Cases 1a and 6a, respectively). A more‐tilted Mars (34.64°) produced lower WEP values than at the current obliquity (25.19°). (e.g., Cases 1a and 2a, respectively). Lastly, when comparing current obliquity (25.19°) to a significantly more‐tilted Mars (54.61°) there was a large increase in WEP values (e.g., Cases 1a and 4a, respectively). Changes in *L*_*s*_ of perihelion (*ϖ*) had little to no effect on WEP values in our study regions.

In summary, the modeling cases with consistently higher WEP values when initializing the MGCM with a global mean surface pressure of 7 mbars were 4a (**54.61°, *ϖ* = 251°, *p*_*sf*_ *=* 7 mbar**) and 5a (**54.61°, *ϖ* = 71°, *p*_*sf*_ *=* 7 mbar**). Modeling cases with consistently higher WEP values when initializing the MGCM with a global mean surface pressure of 14 millibars were 4b (**54.61°, *ϖ* = 251°, *p*_*sf*_ *=* 14 mbar**) and 5b (**54.61°, *ϖ* = 71°, *p*_*sf*_ *=* 14 mbar**) (Figure 13).

### 4.3 Regional Differences in Annual WEP and TI

Regional WEP differences can be observed by examining any of the stacked histogram plots shown in Figures 6, 7, 8, 9, 10, 11 or the intercrater plains comparison plot in Figure 13. It is important to note that slope thresholding and surface unit sub‐setting led several regions and their units to have a reduced number of pixels/bins for analysis. This is particularly the case for the Northeast Syrtis Major Planum region, and for intracrater plains at all of our selected regions. We can observe that the trends in the distributions seen at each region are consistent when comparing them between different calculated cases. Noachis Terra 2 (Figures 6, 11 and 13) and the Jezero MFU unit (Figure 13) consistently have the lowest WEP values in all climate states and Eridania Planitia has the highest WEP values in all cases. Generally, regions containing bedrock do not show clear increases in WEP compared to those without bedrock. However, it is notable that Noachis 2 lacks clear bedrock exposure and also exhibits the lowest WEP across all modeled cases (Figure 13). This observation is discussed further in Section 5.

**Figure 14:**
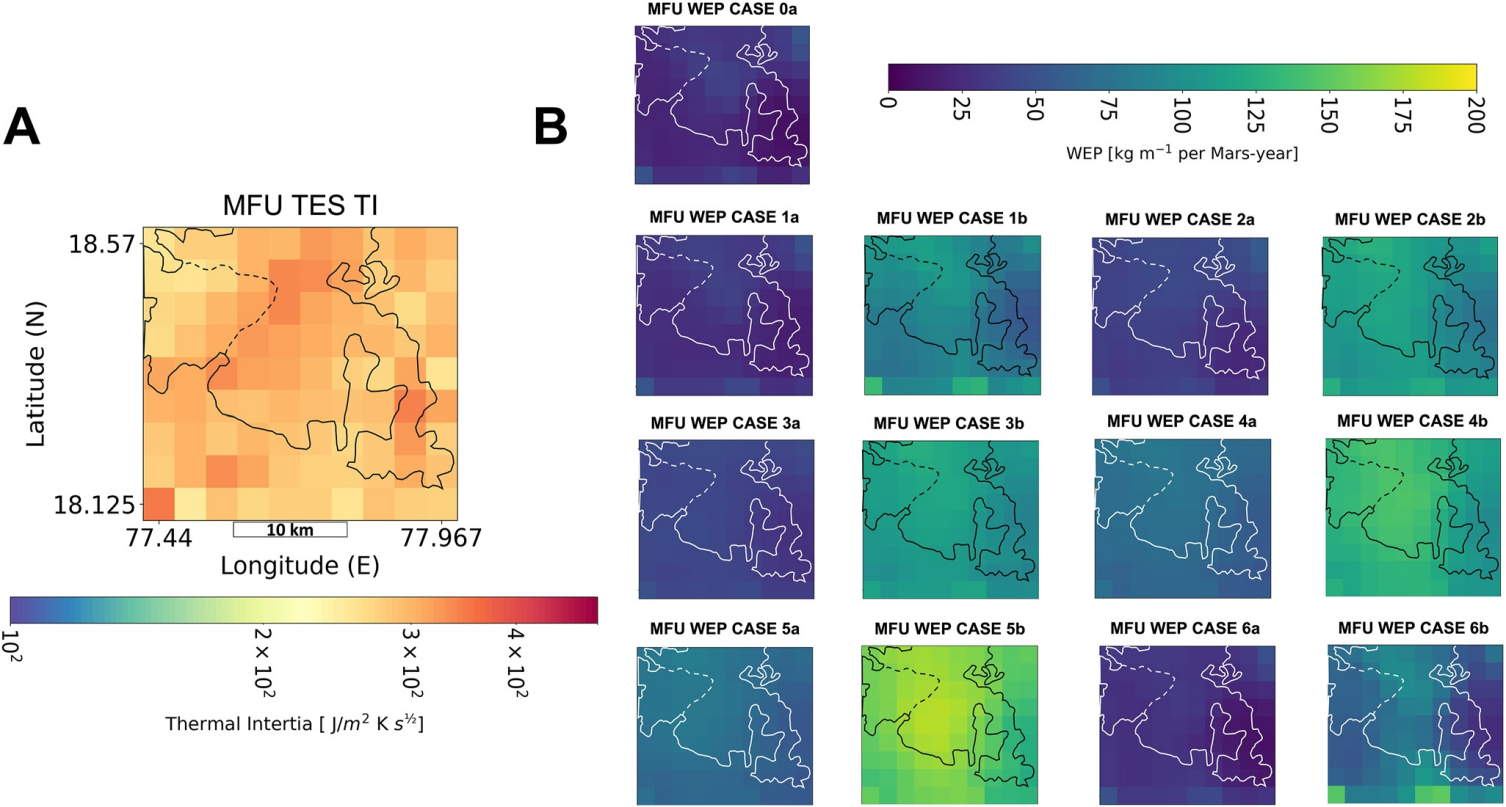
(a) Thermal Emission Spectrometer TI spatial distribution of the Jezero crater floor. (b) Mars Regional Atmospheric Modeling System (MRAMS) annual Wind erosion potential (kg m^−1^ per Mars‐year) spatial distribution on the Jezero crater floor for all 13 modeling cases. Coordinates are the same MRAMS revised tracking as 14A. Black/white outlines indicate areas delineated as mafic floor unit (MFU) by Goudge et al. (2015). Dashed lines indicate separation of rough and smooth units as shown in Stack et al. (2020).

When observing the differences in annual WEP and TI for geologic units, intracrater plains exhibit consistently lower annual WEP than intercrater plains (Figures 6, 11 and 14). Yet for most regions, intracrater TI is similar to or slightly higher than intercrater TI (Figure 5). For bedrock, which has higher TI (Figure 5), WEP values were lower than inter/intracrater plains except for in Eridania Planitia and Terra Sirenum (Figures 6, 11 and 14). Finally, Hesperian lava plains have higher WEP values than intercrater plains in their respective regions, with no consistent difference or trend in TI relative to other units (Figure 5).

We also examined pixel‐by‐pixel intraregional comparisons of annual WEP and TI to determine whether there is a relationship between wind activity and TI. This approach is complementary to the categorical comparisons of WEP within mapped units described previously. Pearson correlation coefficients were calculated for each subunit in each region; all coefficients were less than ±0.5 with the exception of intracrater plains (Tables S2–S11 in Supporting Information S1). However, intracrater plains had low numbers of pixels (∼<10% of total pixels) after slope thresholding, suggesting that the coefficients found for these are not reliable representations for the unit. Generally, there is no predictive linear relationship between WEP and TI for the calculated cases. This suggests that wind erosion is not solely responsible for the spatial distribution of bedrock or coarse material at these regions.

The Jezero MFU consistently had the lowest WEP values except for Noachis 2, which had similar values. This is consistent with our previous observation that intracrater plains generally exhibit lower WEP values. Despite the low WEP relative to the other sites, Jezero exhibits evidence for extensive aeolian erosion (e.g., Day & Dorn, 2019; Fassett & Head, 2005; Schon et al., 2012) and high modern sediment fluxes (Chojnacki et al., 2019). To investigate whether localized wind activity is a factor in the previously reported TI differences across this unit (Ahern et al., 2021; Stack et al., 2020; Warner et al., 2020) the spatial distribution of both TES TI and WEP are shown for the Jezero MFU in Figure 14. The Pearson's correlation coefficients between TES TI and WEP are all less than 0.19 (Table S12 in Supporting Information S1), though we note that it is a small population with only ∼44 pixels. In summary, no clear relationship between TI and WEP is observed, suggesting that variable aeolian activity is not the primary factor in spatial variations in TI. WEP spatial distributions for the MFU are similar across all climate states, with higher values in the northwestern portion of the unit and a decrease toward the southeast (Figure 14b). There are no distinct WEP patterns between the rough crater floor unit and the smooth crater floor unit (Stack et al., 2020).

## 5 Discussion

### 5.1 Role of Amazonian Wind Activity in Bedrock Exposure

The simulated cases used in this study only cover 250 Myr of Mars' climatic past. We acknowledge that this cannot serve to characterize the entirety of Mars' past climate. There are currently no verified atmospheric and orbital characteristics beyond the established timeline in Laskar et al. (2004). In the event that new findings describe the planet's climate further into the past it would be advisable to explore possible changes and contrast to findings in this study.

**Figure 15:**
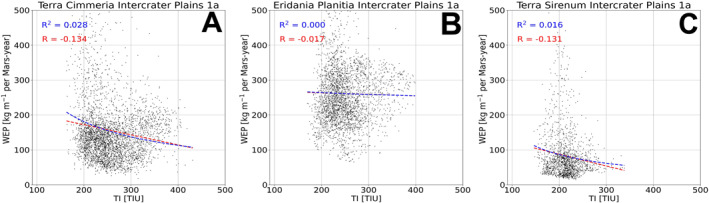
Scatter plots of annual Wind erosion potential (WEP) (kg m^−1^ per Mars‐year) versus TI for each spatial bin for three example locations. Red dashed lines are linear regressions and the corresponding red “R” value is the Pearson correlation coefficient. Blue dashed lines are power law fits, with the blue R‐squared value indicating goodness of fit. (a) Scatter plot of intercrater plains at Terra Cimmeria for case 1a. (b) Scatter plot of intercrater plains at Eridania Planitia for case 1a. (c) Scatter plot of intercrater plains at Terra Sirenum for case 1a. Similar correlation coefficient calculations for the remaining regions are found in the Supporting Information S1 (Tables S1–11). There is no direct relationship between annual WEP and local TI values for any of our study regions.

Bedrock surfaces do not exhibit elevated annual WEP compared to other surface units in the majority of model cases. Exceptions are found at Eridania Planitia, Terra Cimmeria and Terra Sirenum (for some modeling cases), where the bedrock unit WEP distributions are fully or partially shifted to higher values than other units (Figures 5, 6, 7, 8, 9, 10, 11). However, WEP histograms are broad and/or bi‐modal (Figures 5, 6, 7, 8, 9, 10, 11). Additionally, there also does not appear to be a pixel‐level correlation for WEP and bedrock exposure (using TI as a proxy) within intercrater plains (Figure 15).

**Figure 16:**
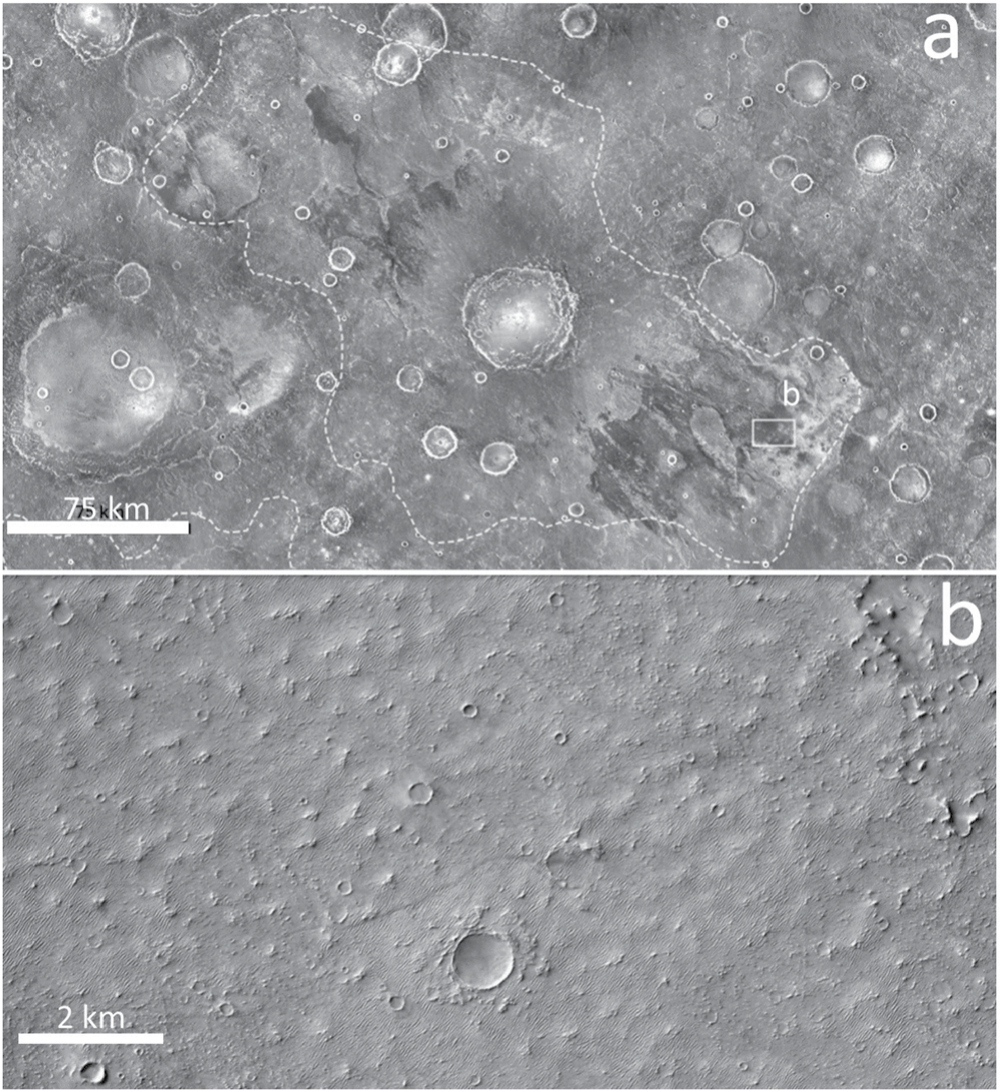
(a) Thermal Emission Imaging System nighttime radiance mosaic; white dashed lines outline lNh units in the Noachis2 region. Darker tones indicate lower thermal inertia material. (b) Portion of Context Camera image D01_027640_1638_XN_16S326W showing ubiquitous bedforms across the surface.

When comparing regions that contain bedrock to regions that do not, there are no consistent differences in annual WEP. Noachis Terra 3 intercrater plains, a region lacking bedrock exposure, has higher WEP values than intercrater plains that do contain bedrock exposures in the Noachis Terra 1, East Hesperia Planum, Terra Cimmeria and Terra Sirenum study regions. However, despite our general finding that there is no consistent relationship between bedrock exposure and WEP values, we note that Noachis Terra 2 is one instance where modeled WEP is consistently low across modeling cases and expanses of bedrock are generally absent. Noachis Terra 2 contains several large, flat late‐Noachian plains units (Figure 1b). High‐resolution imaging suggests the presence of bedrock under a thin sediment cover within these plains (Figure 16). In planform, this region of interest resembles large bedrock exposures observed in other regions (Figure 1b). Thus, we suggest that this is an area where consistently weak Amazonian wind activity may have prevented aeolian removal of fines, leading to lack of bedrock exposure.

Finally, we note that Hesperian volcanic plains, which have thick regolith, have consistently higher annual WEP than other units in their respective regions. This suggests that spatial variation in aeolian activity is not the primary control on bedrock exposure, and supports the hypothesis that differences in material properties are a major factor in the spatial distribution of bedrock exposure, although it may exhibit some control in differences in sediment removal where material properties are otherwise similar.

### 5.2 Implications for Jezero MFU Unit

As shown in previous work, there is strong evidence for aeolian erosion in Jezero crater (e.g., Chojnacki et al., 2019; Fassett & Head, 2005; Schon et al., 2012; Day & Dorn, 2019). Repeat HiRISE observations of dunes in both Jezero crater and Syrtis Major also show moderate to high sand flux values compared to other regions on Mars (Chojnacki et al., 2019). Although wind erosion is clearly occurring within Jezero, our models predict relatively low annual WEP on the MFU compared to volcanic plains in Hesperia Planum and Syrtis Major Planum, and do not predict differences in WEP across the “fractured rough” and “fractured smooth” MFU subunit boundary. Thus, our atmospheric modeling does not support the hypothesis that increased vigor in surface processes is solely responsible for increased TI and lack of thick regolith in this region (Section 1).

It is possible that the MRAMS simulations do not adequately predict the local‐scale wind environment or activity at Jezero crater or on crater floors in general (crater floors exhibit reduced WEP compared to surroundings for all regions). However, within large craters such as Jezero, the diurnal temperature cycle would be expected to induce radial slope‐wind circulations that counteract or modulate regional winds which manage to flow over their often formidable rims (Rafkin et al., 2001). This process would thus result in a tendency for weaker winds near the middle of the floor of such craters, thus it is not surprising that our models predict low WEP over flat crater floors. Another consideration is that our WEP models assume infinite sand supply at all study regions. Limitations on sand supply in other regions may reduce effective wind erosion to levels below those experienced within Jezero. An improvement that could be undertaken in future work could be to estimate or parameterize sand supply for better interpretation of WEP results between regions.

Assuming that the comparatively low annual WEP values are correct, and that the MFU is dominated by a competent rock type (Section 1), there are other potential explanations for the full suite of observations and modeling. One possibility is that the MFU was buried, protecting it from impacts and regolith development, and then recently exhumed. The “fractured smooth” part of the MFU might represent remnant material from such an overlying unit, that has undergone more aeolian deflation and stripping toward the east (e.g., Holm‐Alwmark et al., 2021; Warner et al., 2020). Perseverance and Ingenuity have also observed a striking variety and distribution of surface materials on the MFU, providing additional evidence for burial and exhumation (Sun et al., 2022). The first Perseverance drill attempt was into a light‐toned “paver” rock; the drill attempt completely pulverized the rock during the drilling process, which was not observed during pre‐flight drill attempts on any of the rocks tested (Simon et al., 2022). The second Perseverance drill attempt onto target “Rochette” was successful and showed coarse interlocking crystals, suggesting a competent igneous rock very different than the paver stones. The light‐toned, polygonal paver stones could represent an active deflation surface that may have been thicker in the past and prevented comminution and regolith development on the competent material below.

Additional support for the burial/protection hypothesis comes from crater size‐frequency distributions (down to 10 m diameter) (Warner et al., 2020). A ∼1–2 Ga crater retention age is indicated from craters down to ∼70 m, similar to that at the InSight landing site. Below that size, the SFD decreases, well below both the equilibrium function and the regolith‐covered lava plains at InSight (Warner et al., 2020). The SFD suggests that either (a) craters up to 70 m in diameter are actively removed from the MFU faster than they are produced, or (b) a weaker, meters‐thick overlying unit contained the missing small craters prior to its removal to expose the stronger rock of the MFU. Given the crater retention ages of all craters here, this removal process would have occurred in the Amazonian.

Though we lack a quantitative comparison, the apparent MFU surface roughness is notable and visibly distinct from the Gusev and InSight lava plains. It is possible this intrinsic roughness prevents complete obscuration of the competent rock materials by concentrating finer comminuted material into patchy topographic lows instead of a uniform layer. Landscape evolution models and detailed mapping of MFU surface materials could potentially be used to evaluate the role of initial unit roughness on the rate and distribution of regolith development. However, a complicating factor is that the initial unit roughness of the MFU is unknown.

Finally, another possibility is that the MFU unit is dominated by a competent material that breaks down into fine particles capable of saltation, rather than a crystalline igneous material. For example, a welded ignimbrite deposit would be expected to create rocky crater ejecta but also is composed primarily of fine grains, and thus might break down differently than basalts. Though in‐situ imaging shows evidence for interlocking crystals in some samples (Schmidt et al., 2022), the diversity of materials observed at the surface (Sun et al., 2022) suggests that rocks of variable competency might be present.

### 5.3 Effects of Modeling Case on Annual WEP

We found that the greatest effect on annual WEP from different climate states was from an atmospheric pressure increase. When the MGCM was run at an initial global mean atmospheric surface pressure (***p***_***sf***_) of 14 mbars (all “b” states, Figures 5, 6, 7, 8, 9, 10, 11, 12), the annual WEP values at all regions nearly doubled. This is not surprising, as WEP is directly proportional to atmospheric air density (Kok et al., 2012). However, increasing the atmospheric mass also nonlinearly affects the near‐surface winds that are part of the WEP calculation, since more of the available insolation goes into heating the atmosphere rather than kinetic energy of air flows (i.e., winds). This finding is consistent with in‐situ observations at Gale crater, where sand flux was observed to be higher at night (Baker et al., 2022); measured atmospheric air pressure is higher at nighttime (Martinez et al., 2017).

The second property that had the greatest effect on annual WEP values was obliquity (***ε***). We found that the greater the obliquity (i.e., ***ε*** = 54.61°), the higher the annual WEP values it would produce at our lower‐latitude study areas. If a planetary body has a higher tilt, the insolation of its lower‐latitude regions will be more variable throughout the year (e.g., Khavrus & Shelevytsky, 2012). Coupled with the effects of topography and seasonal surface ice, this can potentially significantly alter the wind patterns and magnitudes.

This principle traces well with all modeling cases with the exception of cases 2 and 3 (“a”and “b” cases). These cases have obliquities of 34.64° and yet the annual WEP value averages for all the regions and units contained therein are less than cases 0 and 1 (“a” and “b” cases, ***ε*** = 25.19°) and similar to that of climate states “6a” and “6b” (***ε*** = 14.71°). Haberle et al. (2003) conducted orbital change experiments using the NASA Ames GCM, finding a similar dip in wind stress at an obliquity of 30°. They found that while winter baroclinic storms enhanced wind stresses at a low obliquity, and intensified Hadley return flow increased wind stresses at high obliquity, both of these flows were relatively weak at 30°. This effect likely explains the low WEP values observed in cases 2 and 3. The similarly low WEP observed in case 6 may be a result of the low latitude of our regions of interest, with the enhanced baroclinic activity found by Haberle et al. (2003) occurring primarily at higher latitudes.

Finally, when it comes to solar longitude of perihelion (***ϖ***), there is no significant effect on WEP values with either 251° (current seasonal cycle) or 71°(reversed seasonal cycle). Thus, seasonal cycles due to axial tilt variations are not a significant factor controlling the wind patterns of Mars' atmosphere at our study regions in the late Amazonian and have little effect on annual WEP values.

## 6 Conclusions

We used the MRAMS atmospheric model to predict WEP at 10 different highlands locations, including the Jezero MFU to assess the relative role of wind in creating the present‐day spatial distribution of exposed bedrock or other high TI surfaces. We varied the climate states of the model to investigate how WEP varies with obliquity, Ls of perihelion, and mean atmospheric pressure. We also examined how WEP varies across different terrain types, including intracrater plains, intercrater plains, and Hesperian volcanic units. From our analyses we have drawn the following conclusions:

1. There is no observed relationship between WEP and bedrock exposure, supporting the hypothesis that spatial variations in material properties are the dominant control on where bedrock is found.
2. Flat‐lying surfaces within craters consistently have lower WEP values than intercrater plains and Hesperian volcanic units.
3. Of all regions studied, the Jezero MFU had the lowest modeled WEP, characteristic of other intracrater plains units. Because the values are low compared to other known volcanic plains (e.g., Syrtis Major), and because the MFU has very little regolith cover compared to known volcanic plains, this suggests that additional factors besides strength of surficial processes must account for these differences. Burial/exhumation, intrinsic surface roughness, and/or differences in lithology are potential causes discussed in Section 5.2.
4. Increased atmospheric pressure had the most drastic effect on annual WEP values, where doubling the pressure increased the values by a factor of ∼1.7 on average.

## Supporting information

Supporting Information S1

## Data Availability

The THEMIS IR images (Christensen, 2002), TES TI maps (Putzig et al., 2009), MOLA elevation data (Smith et al., 2003) and Context Camera images (Malin, 2007) used in this work can be found in the Planetary Data System Geosciences Node. Software and data used for this analysis can be found in a permanent zenodo repository (Gary‐Bicas et al., 2022a, 2022b). The software used for processing datasets was Python version 3.8 (Van Rossum & Drake, 2009). The analysis was divided between four different virtual workbooks known as “Jupyter” notebooks. The data were analyzed using both Pandas (McKinney, 2010) and Numpy (Harris et al., 2020) modules where data was ingested into dataframes and arrays. When binning the datasets for comparison and doing statistical analysis, we utilized the Scipy module (Virtanen et al., 2020). Additionally, the Matplotlib module (Hunter, 2007) was used for plotting and creating figures.
