# Supplementary material for: Investigating the Role of Amazonian Mesoscale Wind Patterns and Strength on the Spatial Distribution of Martian Bedrock Exposures
Source: J Geophys Res Planets. 2022 Nov 18;127(11):e2022JE007496. doi: 10.1029/2022JE007496 (PMC10078484; doi:10.1029/2022JE007496)
Supplement: Supplementary file 1 — Supporting Information S1 [file JGRE-127-0-s001.docx]

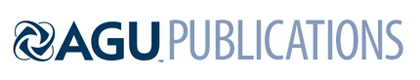


*Journal of Geophysical Research Planets*

Supporting Information for

**Investigating the role of Amazonian mesoscale wind patterns and strength on the spatial distribution of Martian bedrock exposures**

C. E. Gary-Bicas^1^, T. I. Michaels^2^, A. D. Rogers^1^, L. K. Fenton^2^, N. H. Warner^3^, and A. C. Cowart^1^

^1^Department of Geosciences, Stony Brook University, Stony Brook, NY, 11790, United States

^2^ Carl Sagan Center, SETI Institute, 189 Bernardo Ave Suite 200, Mountain View, CA 94043, United States

^3^ Department of Geological Sciences, State University of New York at Geneseo, Geneseo, NY 14454, United States

**Contents of this file**

Introduction

Figures S1 to S10

Tables S1 to S12

**Introduction**

The following data is organized to give a preliminary view of the regions seen in the manuscript “Investigating the role of Amazonian mesoscale wind patterns and strength on the spatial distribution of Martian bedrock exposures” (Gary-Bicas et al., 2022). Each page contains one of the ten designated regions analysed. Seen at each figure is a Thermal Emission Imaging System (THEMIS) daytime temperature map followed by an overlay of bedrock exposures found by Cowart et al., 2019. Subsequently, a depiction of the region using USGS geologic map #3292 (Tanaka et al., 2014) shows the different geologic units contained therein. We then show a Thermal Emission Spectrometer (TES) thermal inertia (TI) map (Putzig and Mellon, 2007; Putzig and Mellon, 2005) of the same region. Lastly, we show a map depicting Wind Erosion Potential (WEP) values simulated case 1a using the Mars Regional Atmospheric Modeling System (MRAMS, Rafkin et al., 2001). At the bottom of these mapped regions we show a correlation coefficient table that shows the Pearson correlation coefficient between TES TI and Wind Erosion Potential (WEP, Kok et al., 2012) for our delineated units at each region for each of the 13 simulated climate states analyzed using the Mars Global Circulation Model (MGCM, Haberle et al., 1993) and MRAMS.

We refer to the following table for USGS map #3292 for acronyms used in the figures.

| Acronym found in USGS map #3292 | Geologic unit name |
| --- | --- |
| eNh | Early Noachian highland nit |
| mNh | Middle Noachian highland Unit |
| lNh | Late Noachian highland |
| eHv | Early Hesperian volcanic unit |
| AHi | Amazonian and Hesperian impact unit |
| mNhm | Middle Noachian highland massif unit |
| HNt | Hesperian and Noachian transition unit |
| lHt | Late Hesperian transition unit |
| Nhe | Noachian highland edifice unit |

Table S1: USGS geologic map #3292 units found in study regions.

Noachis Terra 1


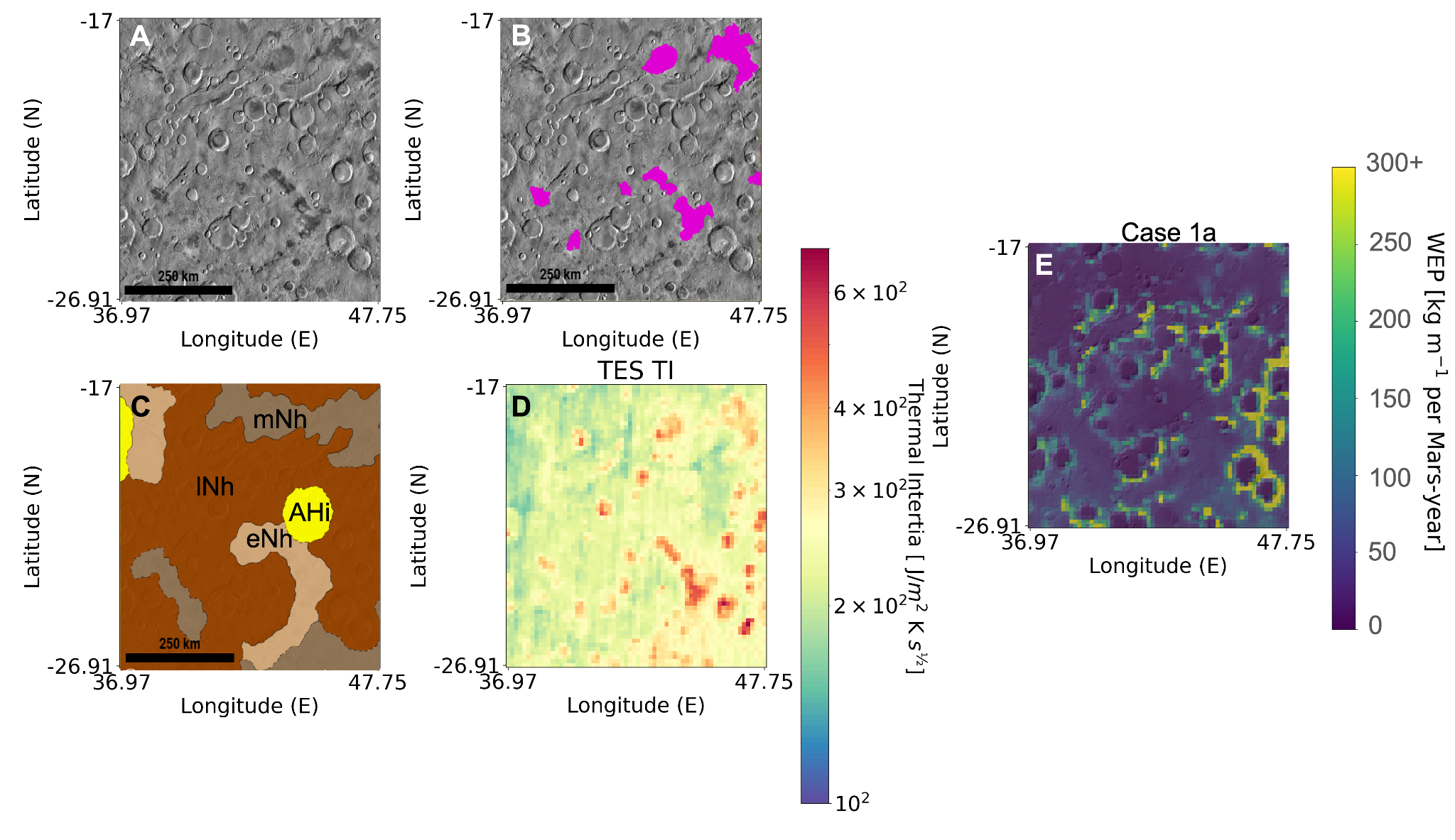


Figure S1: Different views of the Noachis Terra 1 region of study. A) THEMIS Day IR view of the region. B) THEMIS Day IR view of the region overlain by bedrock exposures designated by Cowart et al., 2019 (pink polygons). C) USGS geologic unit map #3292 of the region (Tanaka et al. 2014), labels indicate unit name (see **Table S1**). D) TES TI mosaic of the region (Putzig and Mellon, 2007). E) Spatial distribution of MRAMS WEP values for calculated **case 1a** in the region overlain over MOLA shaded relief.


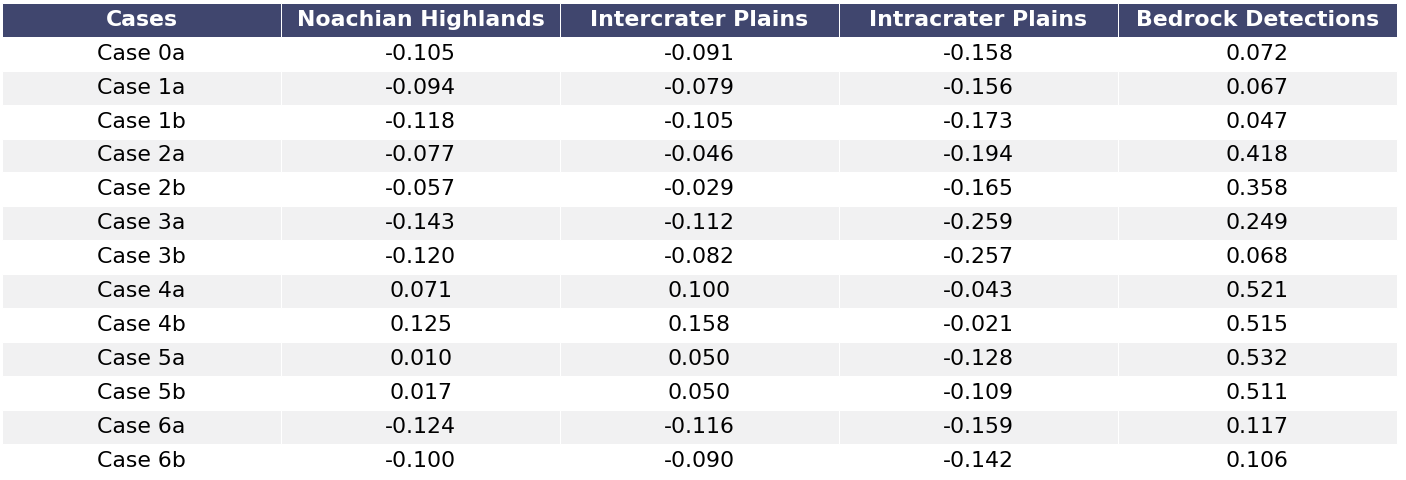


Table S2: Pearson correlation coefficients between MRAMS WEP and TES TI for each unit in Noachis Terra 1 taking into account all 13 climate cases. (Flat lying units i.e. low slope).

Noachis Terra 2


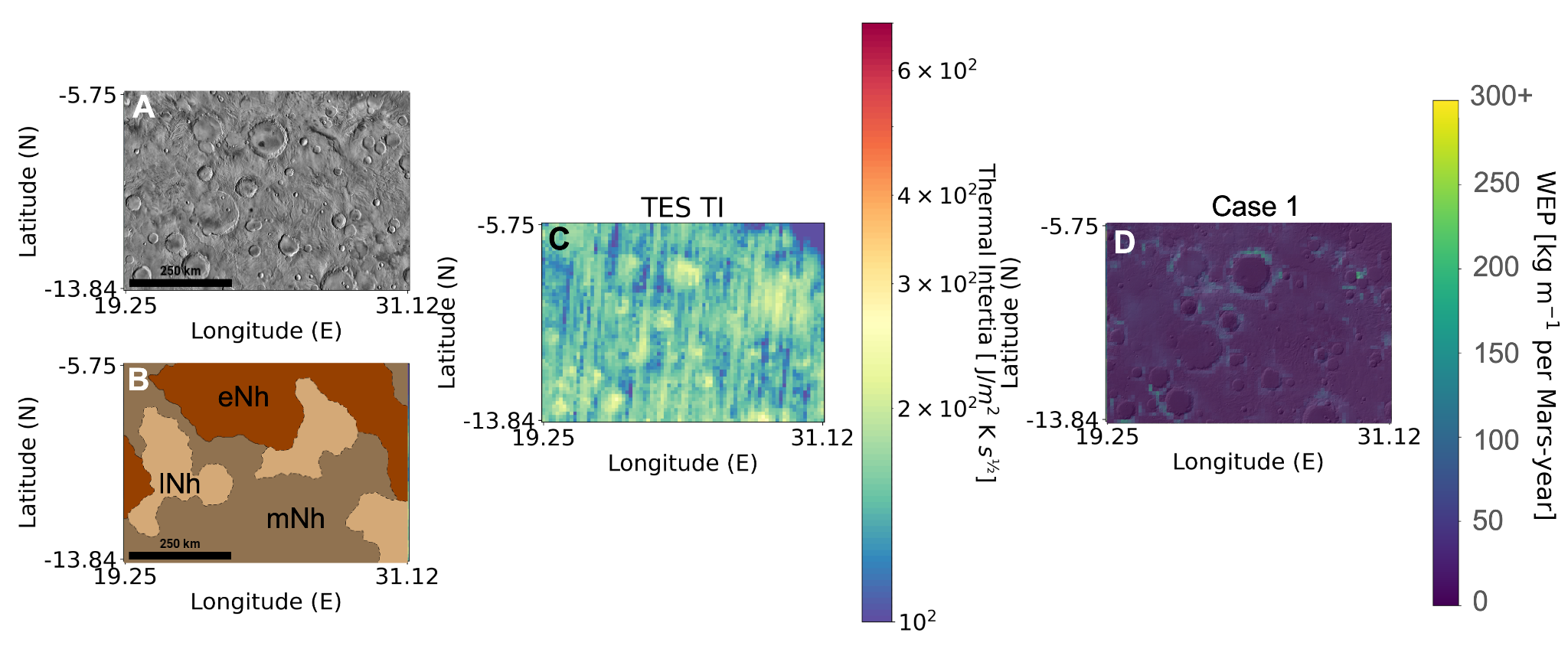


Figure S2: Different views of the Noachis Terra 2 region of study. A) THEMIS Day IR view of the region. B) USGS geologic unit map #3292 of the region (Tanaka et al. 2014), labels indicate unit name (see **Table S1**). C) TES TI mosaic of the region (Putzig and Mellon, 2007). D) Spatial distribution of MRAMS WEP values for calculated **case 1a** in the region overlain over MOLA shaded relief.


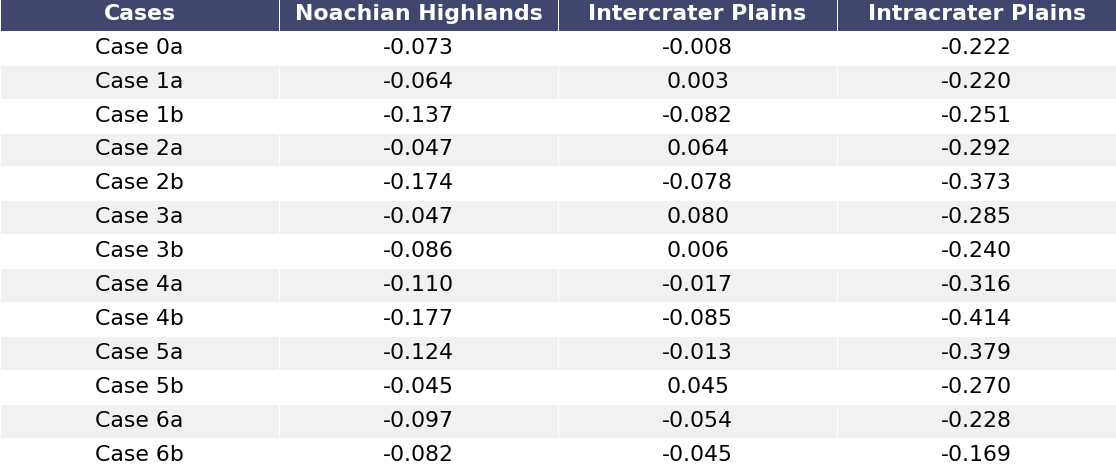


Table S3: Pearson correlation coefficients between MRAMS WEP and TES TI for each unit in Noachis Terra 2 taking into account all 13 climate cases. (Flat lying units i.e. low slope).

Noachis Terra 3


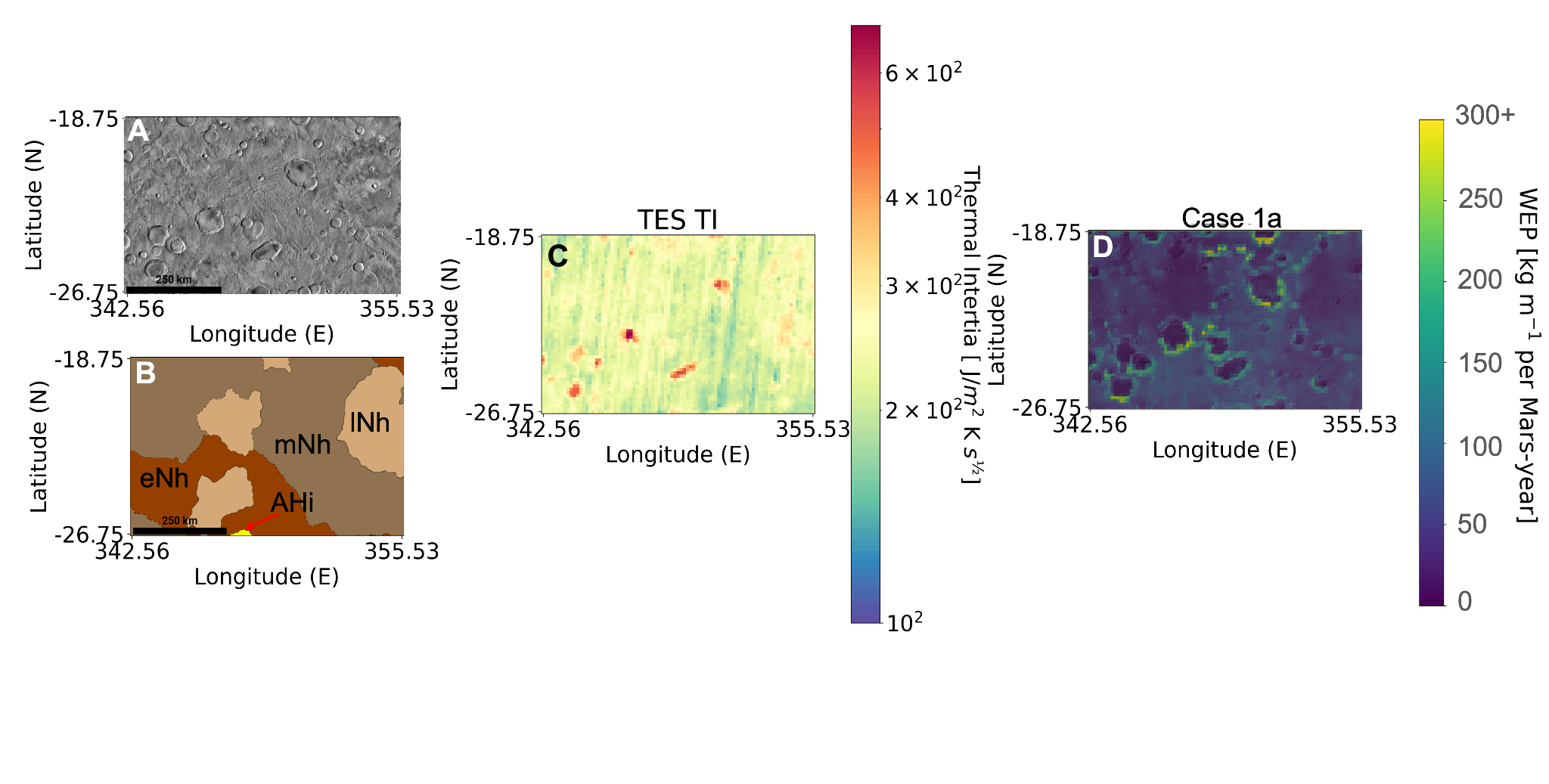


Figure S3: Different views of the Noachis Terra 3 region of study. A) THEMIS Day IR view of the region. B) USGS geologic unit map #3292 of the region (Tanaka et al. 2014), labels indicate unit name (see **Table S1**). C) TES TI mosaic of the region (Putzig and Mellon, 2007). D) Spatial distribution of MRAMS WEP values for calculated **case 1a** in the region overlain over MOLA shaded relief.


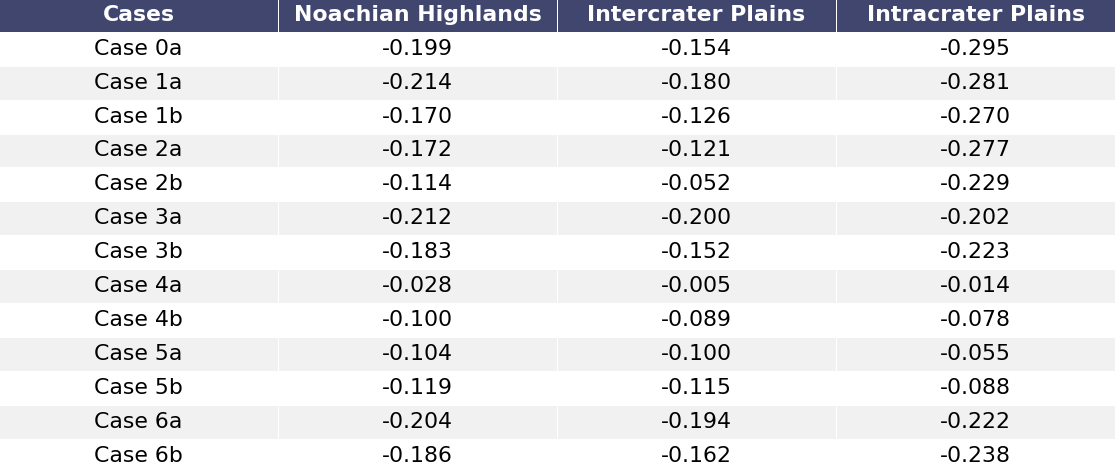


Table S4: Pearson correlation coefficients between MRAMS WEP and TES TI for each unit in Noachis Terra 3 taking into account all 13 climate cases. (Flat lying units i.e. low slope).

South Syrtis Major Planum


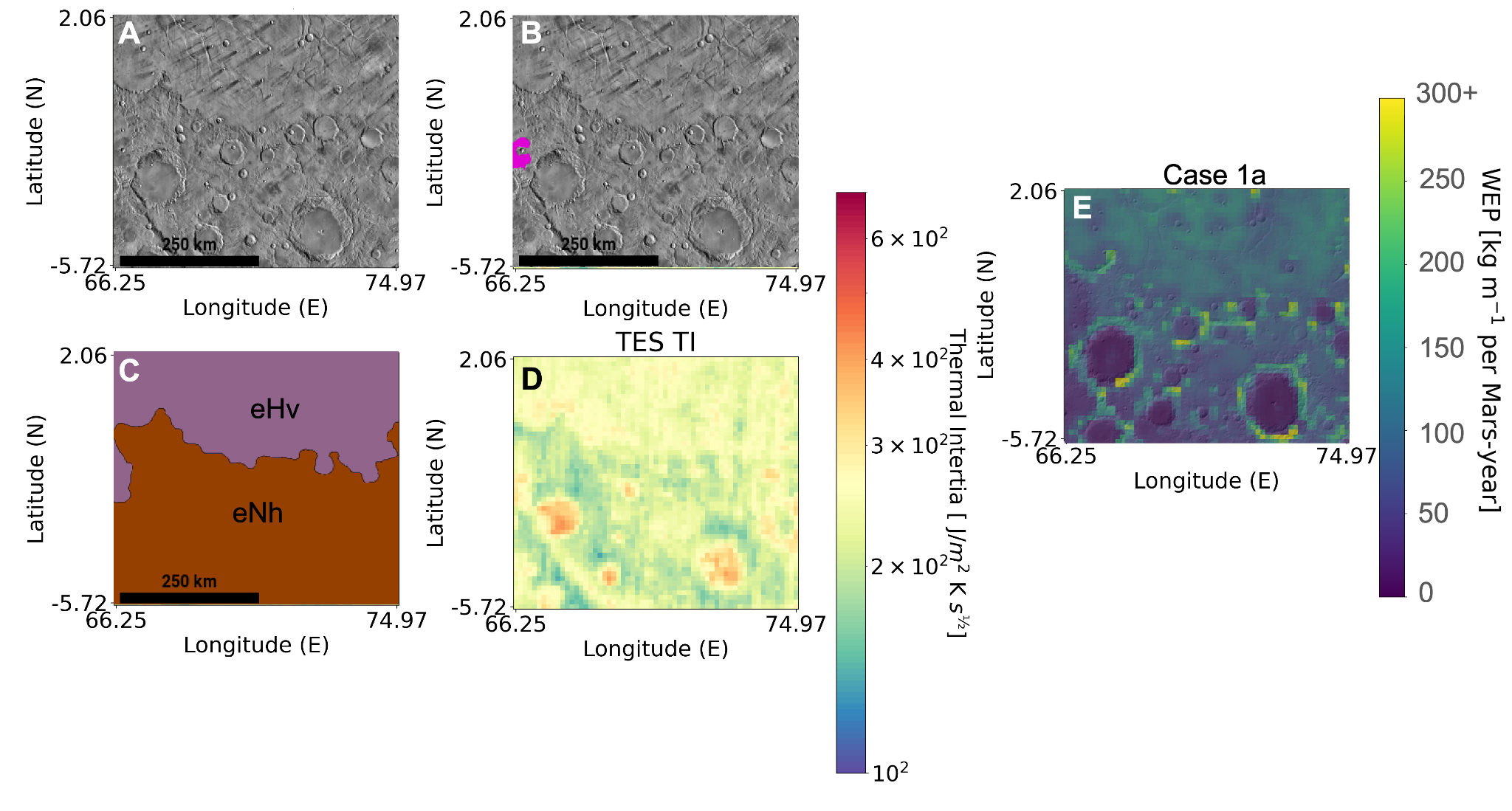


Figure S4: Different views of the South Syrtis Major Planum region of study. A) THEMIS Day IR view of the region. B) THEMIS Day IR view of the region overlain by bedrock exposures designated by Cowart et al., 2019 (pink polygons). C) USGS geologic unit map #3292 of the region (Tanaka et al. 2014), labels indicate unit name (see **Table S1**). D) TES TI mosaic of the region (Putzig and Mellon, 2007). E) Spatial distribution of MRAMS WEP values for calculated **case 1a** in the region overlain over MOLA shaded relief.


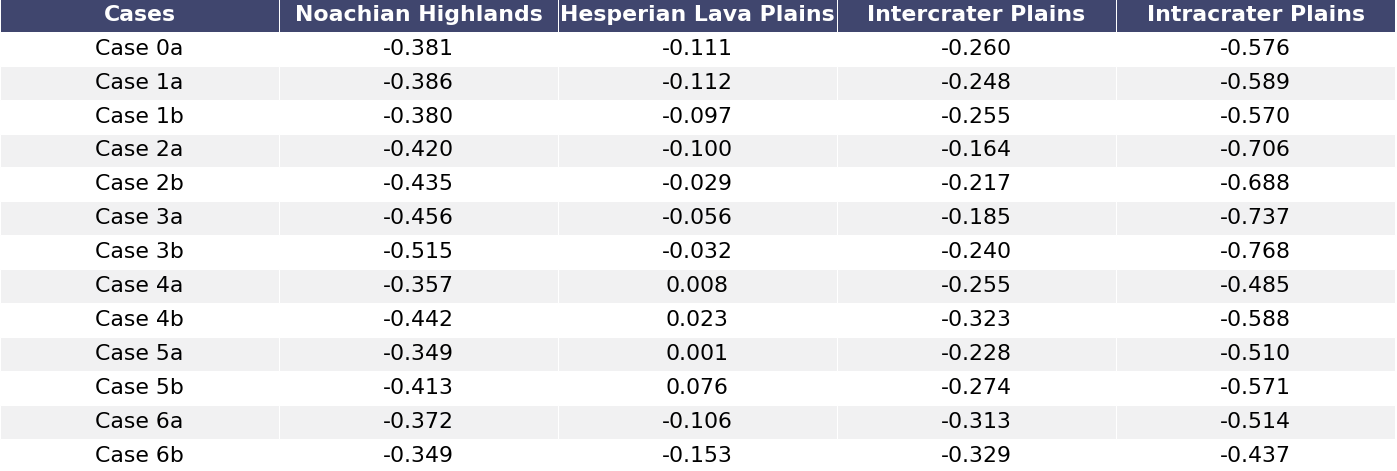


Table S5: Pearson correlation coefficients between MRAMS WEP and TES TI for each unit in South Syrtis Major Planum taking into account all 13 climate cases.(Flat lying units i.e. low slope).

Tyrrhena Terra


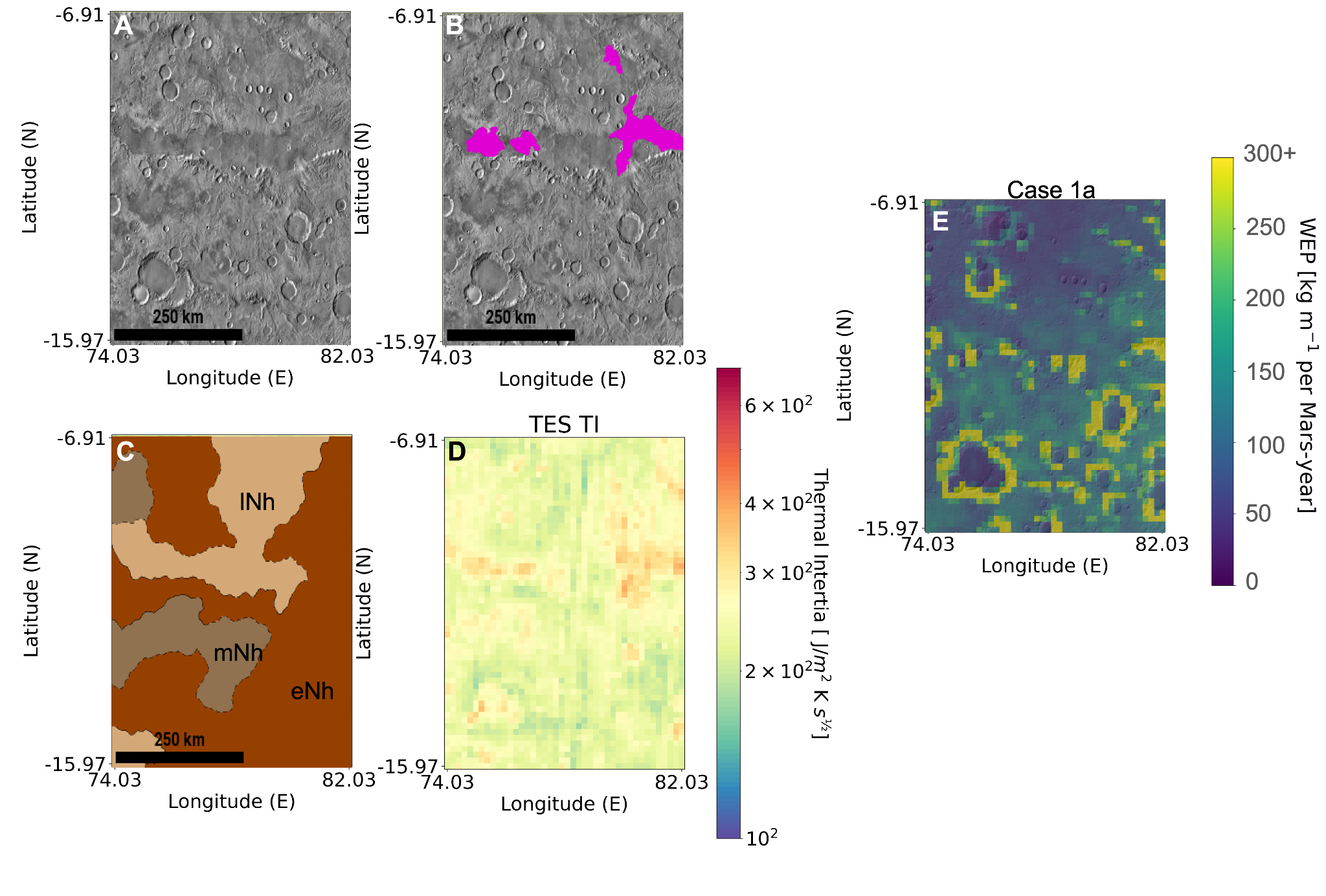


Figure S5: Different views of the Tyrrhena Terra region of study. A) THEMIS Day IR view of the region. B) THEMIS Day IR view of the region overlain by bedrock exposures designated by Cowart et al., 2019 (pink polygons). C) USGS geologic unit map #3292 of the region (Tanaka et al. 2014), labels indicate unit name (see **Table S1**). D) TES TI mosaic of the region (Putzig and Mellon, 2007). E) Spatial distribution of MRAMS WEP values for calculated **case 1a** in the region overlain over MOLA shaded relief.


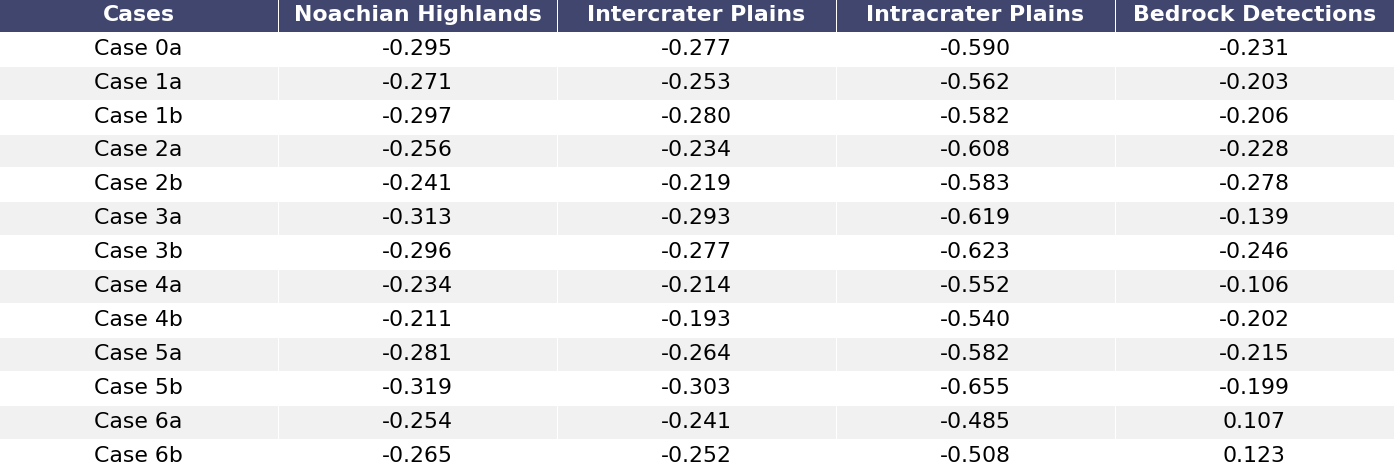


Table S6: Pearson correlation coefficients between MRAMS WEP and TES TI for each unit in Tyrrhena Terra taking into account all 13 climate cases. (Flat lying units i.e. low slope).

Northeast Syrtis Major Planum


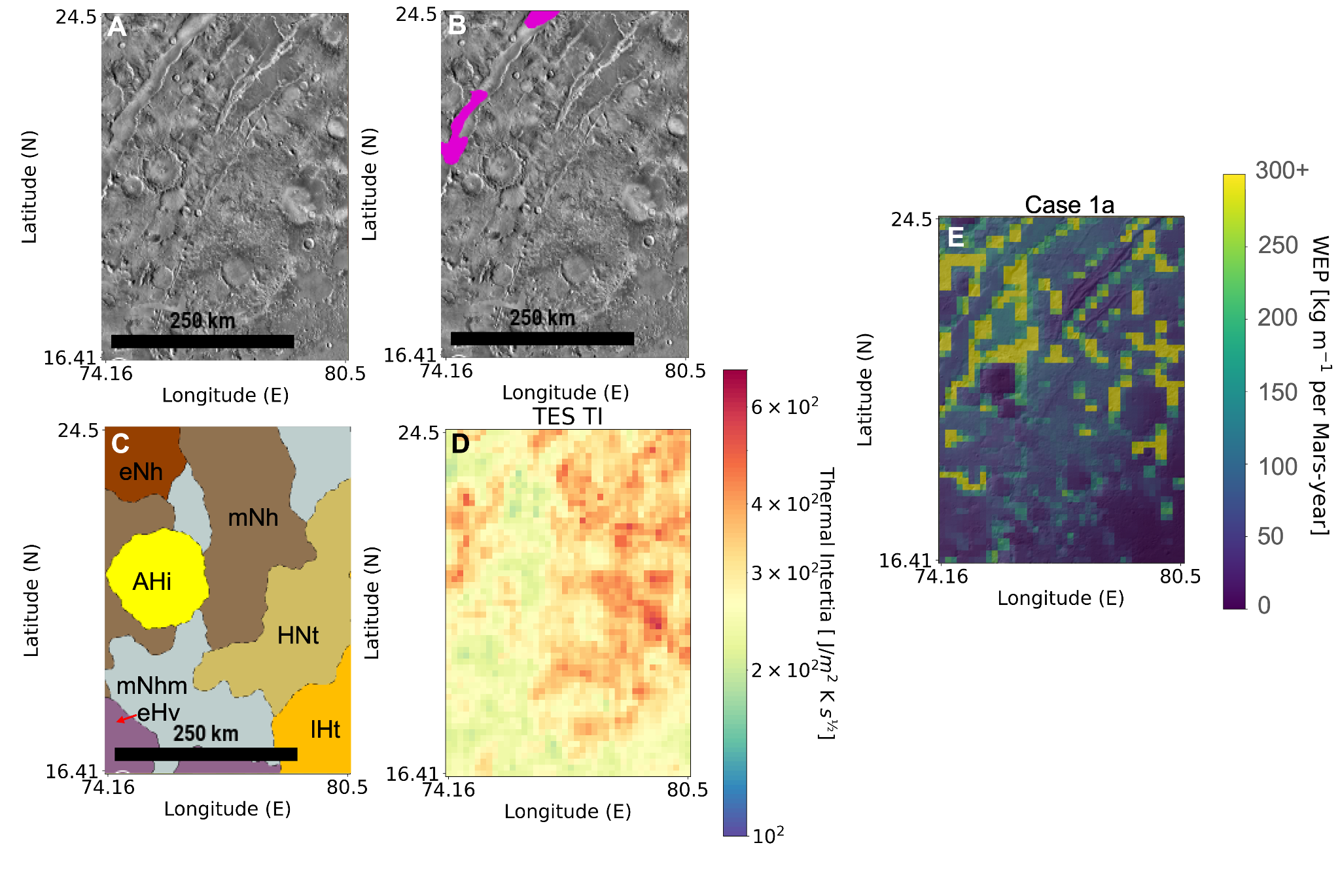


Figure S6: Different views of the Northeast Syrtis Major Planum region of study. A) THEMIS Day IR view of the region. B) THEMIS Day IR view of the region overlain by bedrock exposures designated by Cowart et al., 2019 (pink polygons). C) USGS geologic unit map #3292 of the region (Tanaka et al. 2014), labels indicate unit name (see **Table S1)**. D) TES TI mosaic of the region (Putzig and Mellon, 2007). E) Spatial distribution of MRAMS WEP values for calculated **case 1a** in the region overlain over MOLA shaded relief.


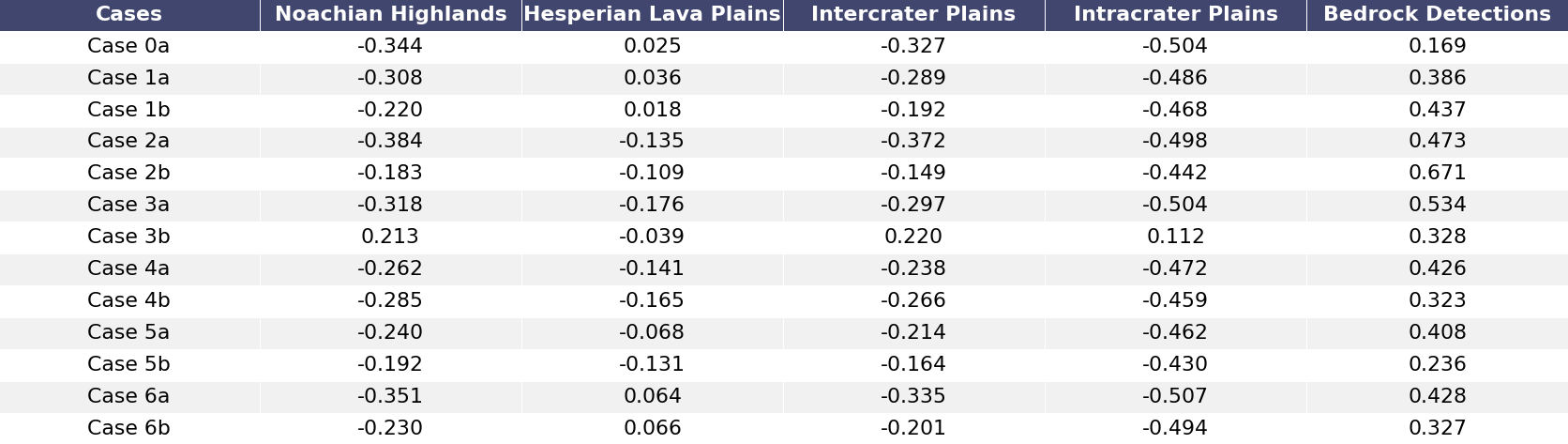


Table S7: Pearson correlation coefficients between MRAMS WEP and TES TI for each unit in South Syrtis Major Planum taking into account all 13 climate cases. (Flat lying units i.e. low slope).

East Hesperia Planum


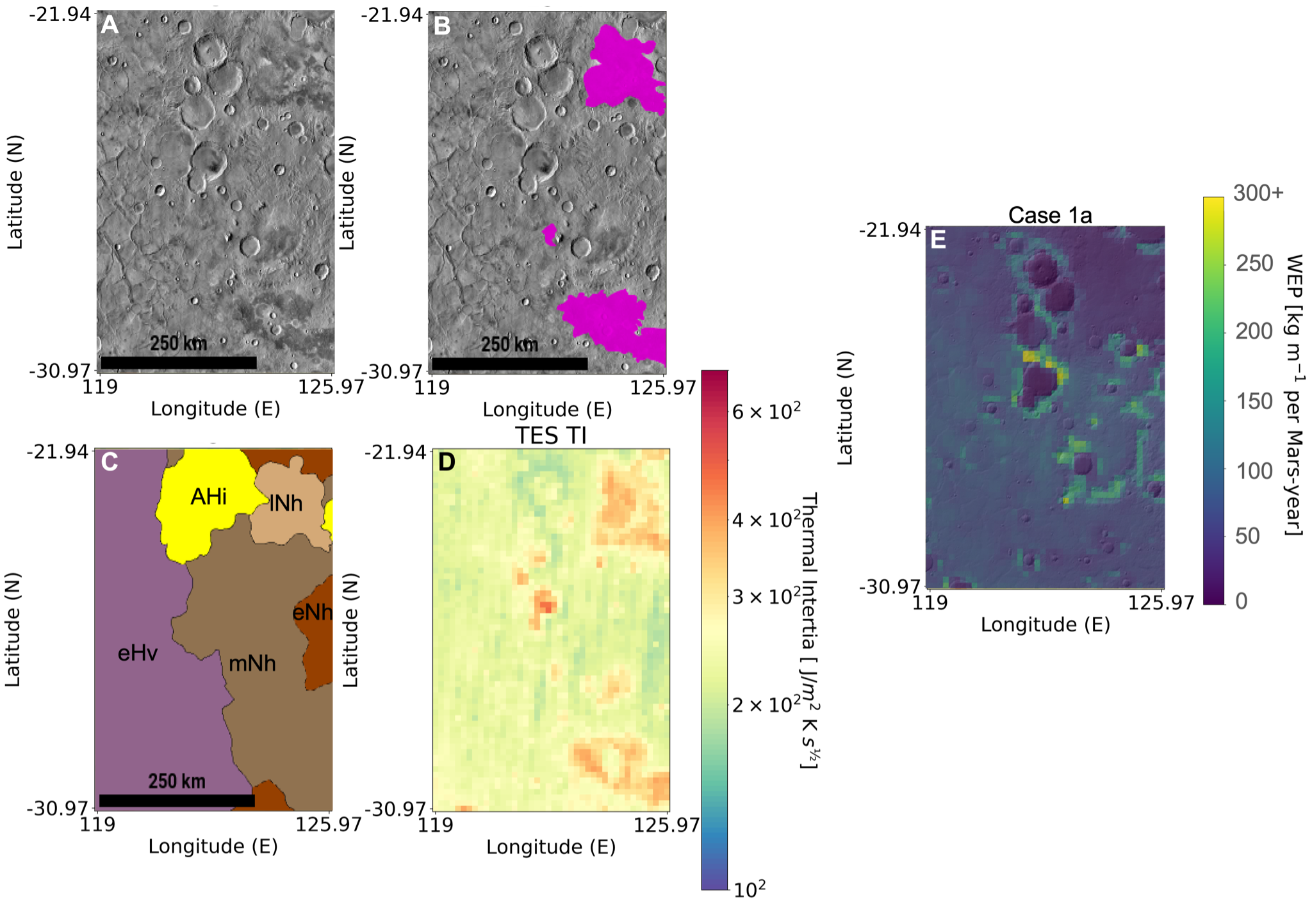


Figure S7: Different views of the East Hesperia Planum region of study. A) THEMIS Day IR view of the region. B) THEMIS Day IR view of the region overlain by bedrock exposures designated by Cowart et al., 2019 (pink polygons). C) USGS geologic unit map #3292 of the region (Tanaka et al. 2014), labels indicate unit name (see **Table S1)**. D) TES TI mosaic of the region (Putzig and Mellon, 2007). E) Spatial distribution of MRAMS WEP values for calculated **case 1a** in the region overlain over MOLA shaded relief.


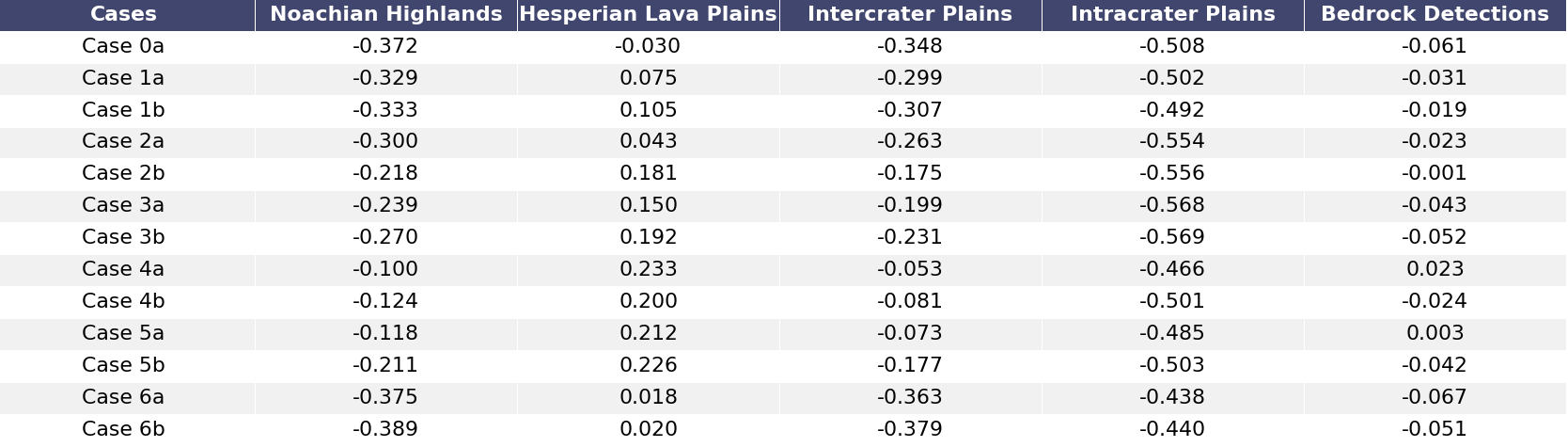


Table S8: Pearson correlation coefficients between MRAMS WEP and TES TI for each unit in East Hesperia Planum taking into account all 13 climate cases. (Flat lying units i.e. low slope).

Terra Cimmeria


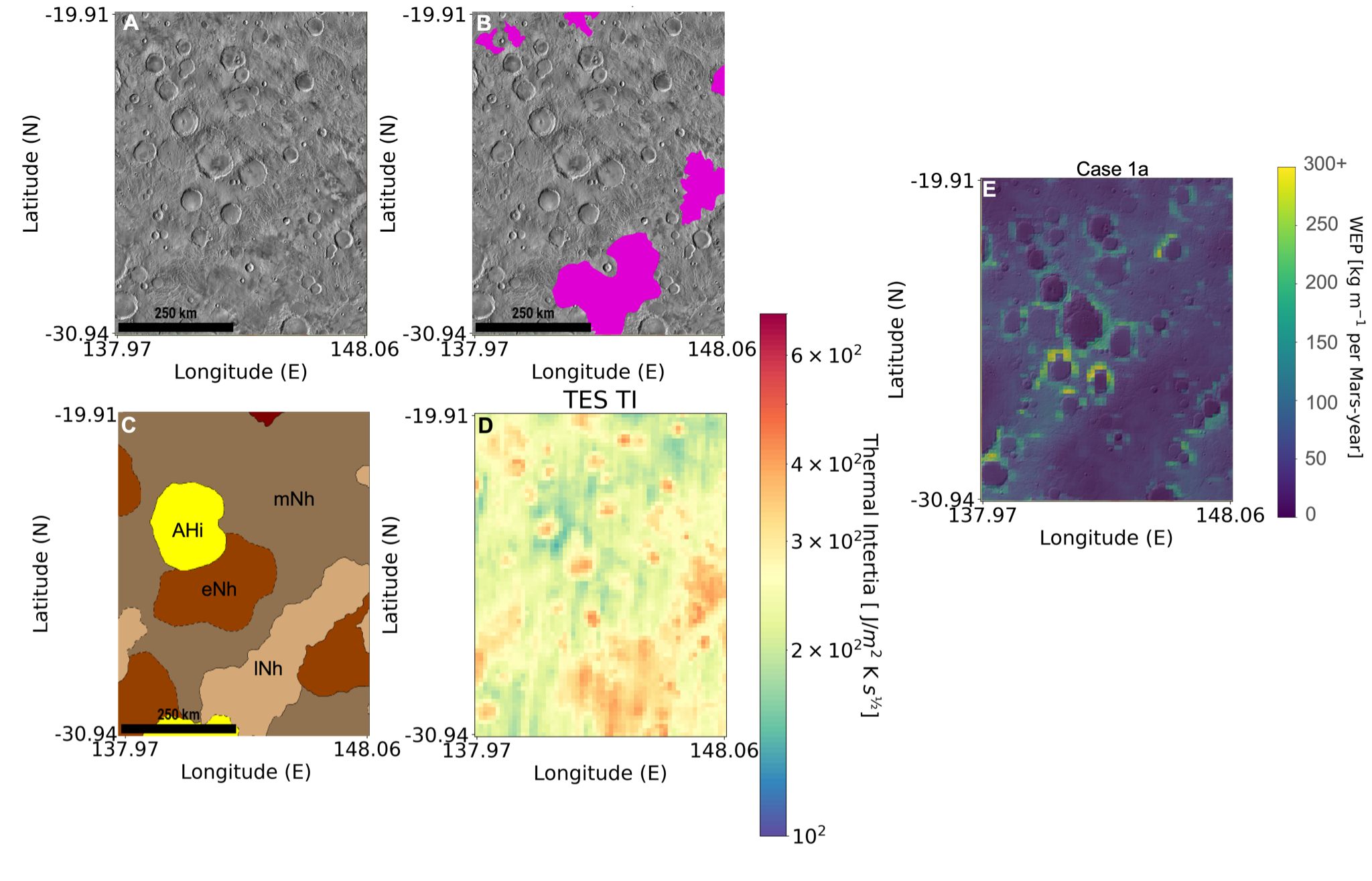


Figure S8: Different views of the Terra Cimmeria region of study. A) THEMIS Day IR view of the region. B) THEMIS Day IR view of the region overlain by bedrock exposures designated by Cowart et al., 2019 (pink polygons). C) USGS geologic unit map #3292 of the region (Tanaka et al. 2014), labels indicate unit name (see **Table S1)**. D) TES TI mosaic of the region (Putzig and Mellon, 2007). E) Spatial distribution of MRAMS WEP values for calculated **case 1a** in the region overlain over MOLA shaded relief.


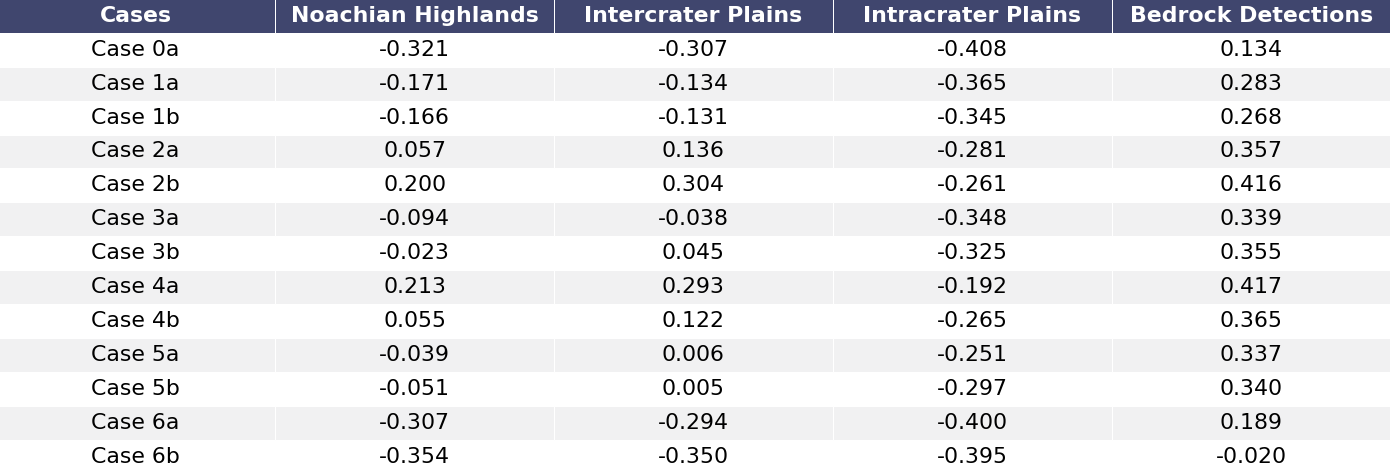


Table S9: Pearson correlation coefficients between MRAMS WEP and TES TI for each unit in Terra Cimmeria taking into account all 13 climate cases. (Flat lying units i.e. low slope).

Eridania Planitia


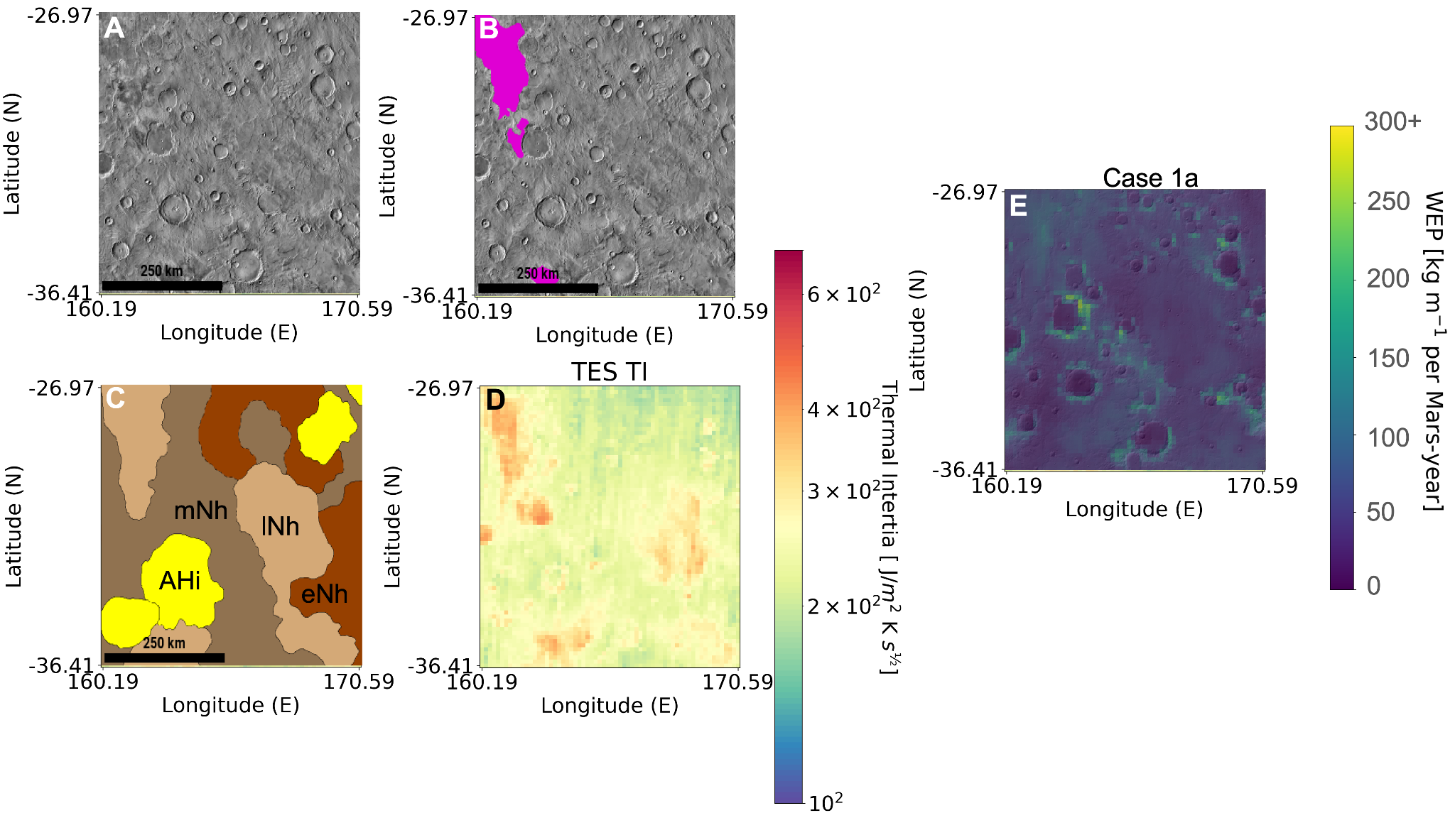


Figure S9: Different views of the Eridania Planitia region of study. A) THEMIS Day IR view of the region. B) THEMIS Day IR view of the region overlain by bedrock exposures designated by Cowart et al., 2019 (pink polygons). C) USGS geologic unit map #3292 of the region (Tanaka et al. 2014), labels indicate unit name (see **Table S1)**. D) TES TI mosaic of the region (Putzig and Mellon, 2007). E) Spatial distribution of MRAMS WEP values for calculated **case 1a** in the region overlain over MOLA shaded relief.


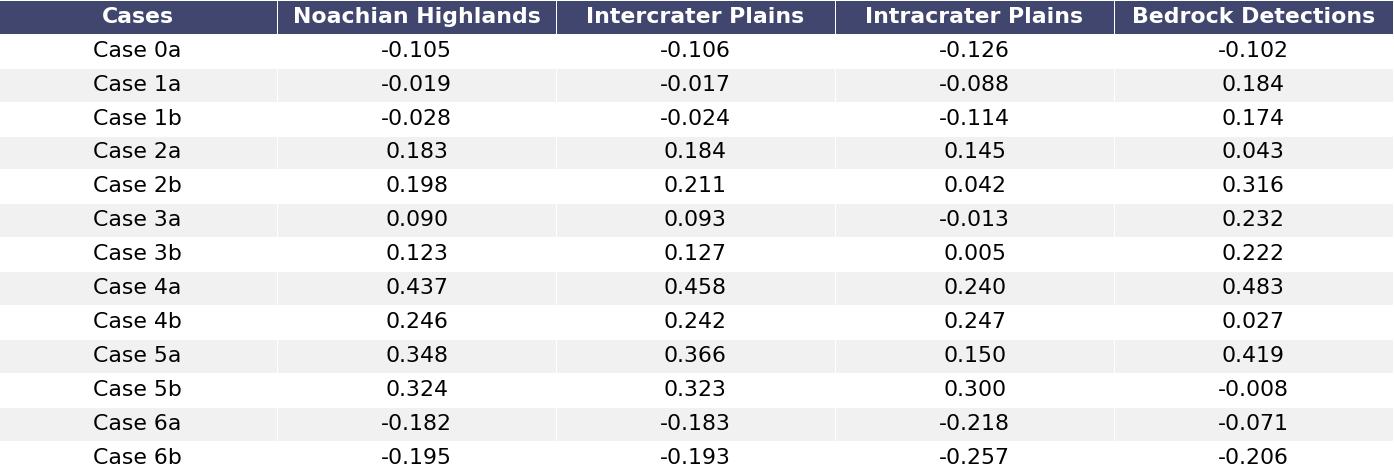


Table S10: Pearson correlation coefficients between MRAMS WEP and TES TI for each unit in Eridania Planitia taking into account all 13 climate cases. (Flat lying units i.e. low slope).

Terra Sirenum


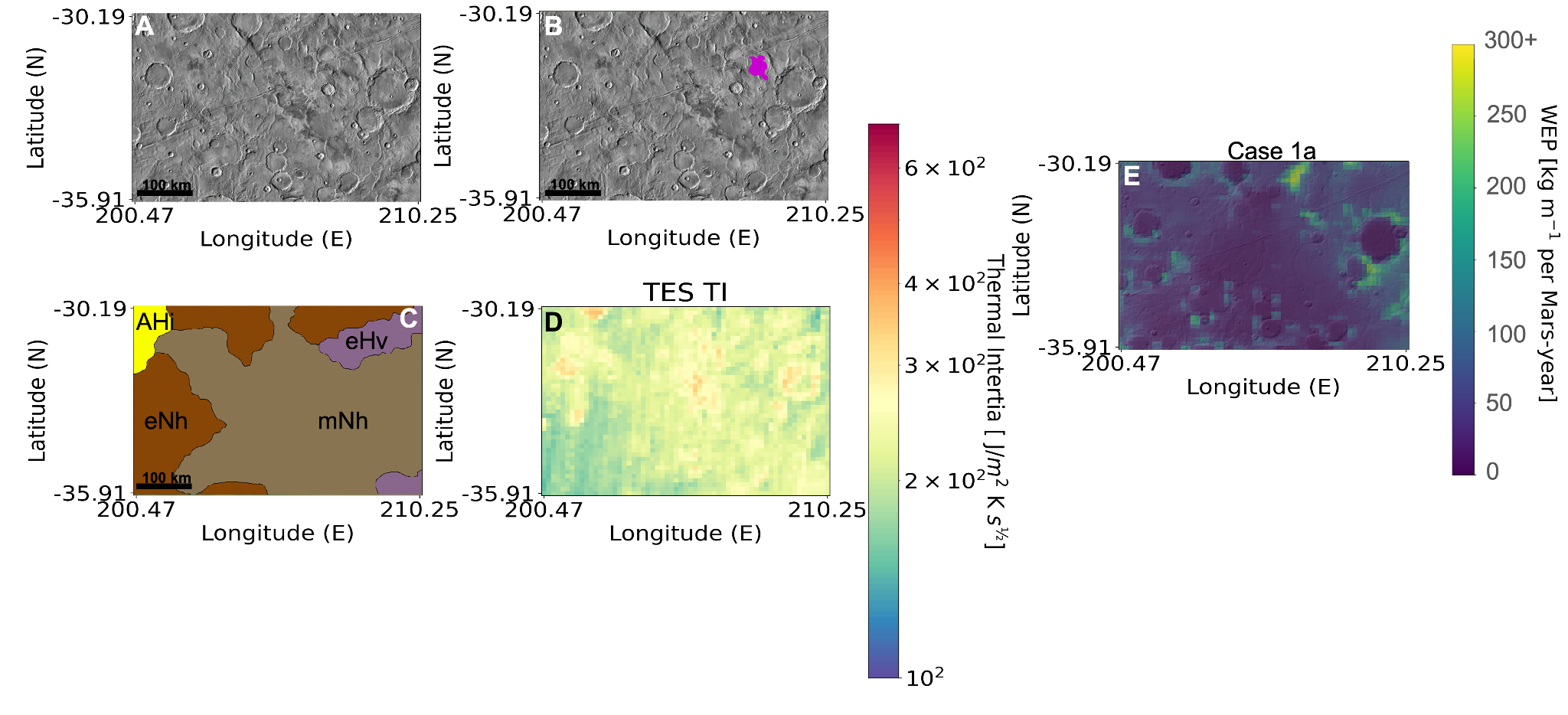


Figure S10: Different views of the Terra Sirenum region of study. A) THEMIS Day IR view of the region. B) THEMIS Day IR view of the region overlain by bedrock exposures designated by Cowart et al., 2019 (pink polygons). C) USGS geologic unit map #3292 of the region (Tanaka et al. 2014), labels indicate unit name (see **Table S1)**. D) TES TI mosaic of the region (Putzig and Mellon, 2007). E) Spatial distribution of MRAMS WEP values for calculated **case 1a** in the region overlain over MOLA shaded relief.


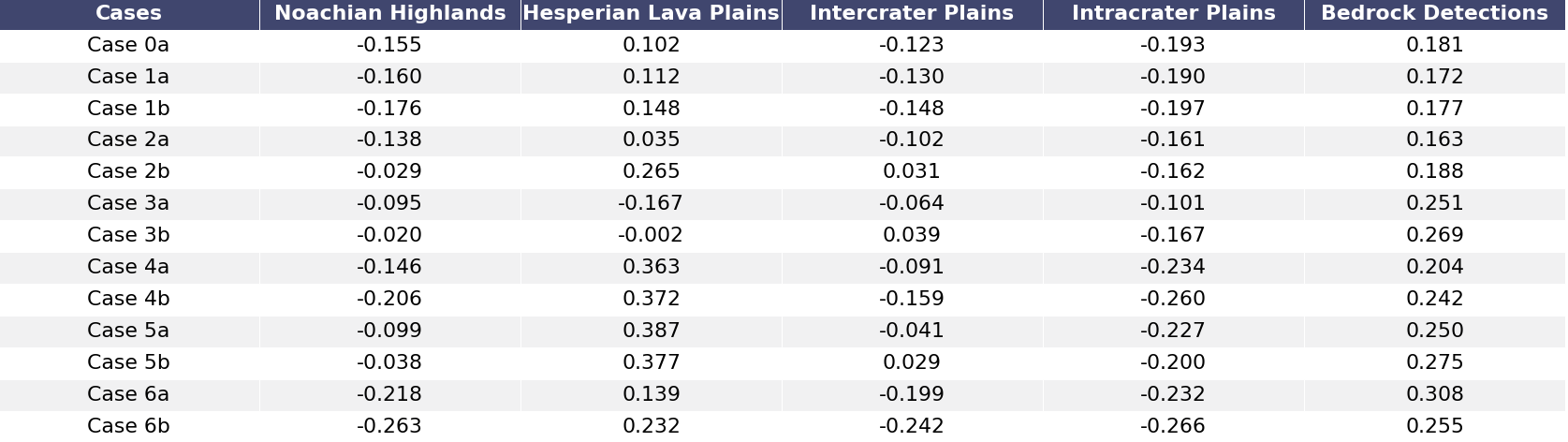


Table S11: Pearson correlation coefficients between MRAMS WEP and TES TI for each unit in Eridania Planitia taking into account all 13 climate cases. (Flat lying units i.e. low slope).


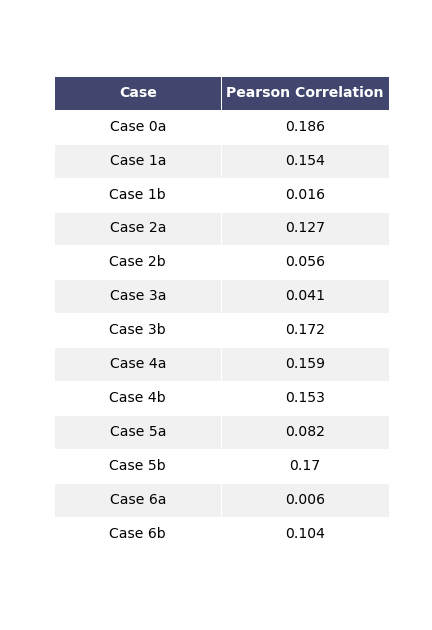


Table S12: Pearson correlation coefficients between MRAMS WEP and TES TI for the MFU at Jezero Crater
